# Desalination Potential of Aquaporin-Inspired Functionalization of Carbon Nanotubes: Bridging Between Simulation and Experiment

**DOI:** 10.1021/acsami.2c03700

**Published:** 2022-06-08

**Authors:** Aysa Güvensoy-Morkoyun, Sadiye Velioğlu, M. Göktuğ Ahunbay, Ş. Birgül Tantekin-Ersolmaz

**Affiliations:** † Department of Chemical Engineering, Istanbul Technical University, Maslak, Istanbul, 34469, Turkey; ‡ Institute of Nanotechnology, Gebze Technical University, Kocaeli, 41400, Turkey

**Keywords:** biomimetic membrane, carbon nanotube, desalination, molecular dynamics, thin film nanocomposite

## Abstract

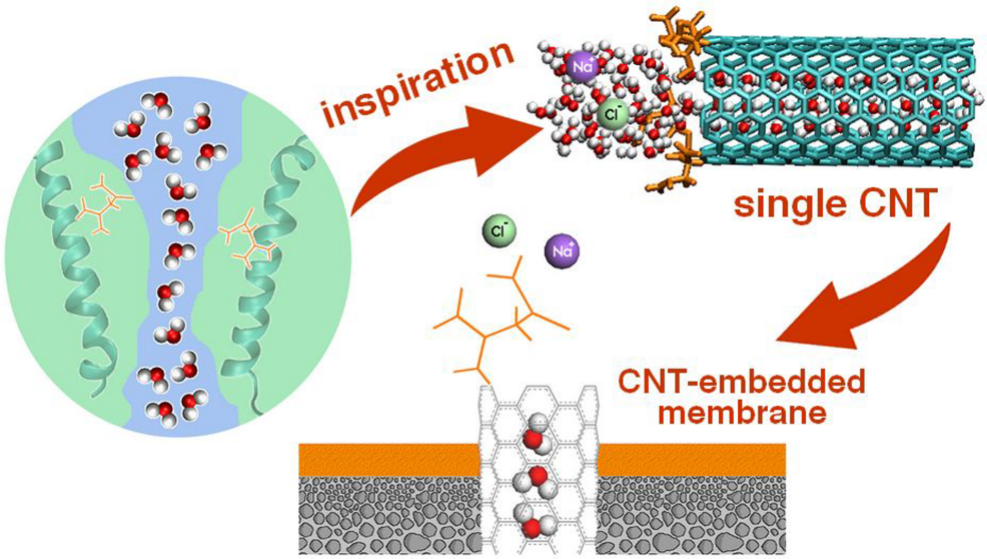

Outstanding water/ion selectivity of aquaporins paves the way for bioinspired desalination membranes. Since the amino acid asparagine (Asn) plays a critical role in the fast water conduction of aquaporins through hydrogen bonding interactions, we adapted this feature by functionalizing carbon nanotubes (CNTs) with Asn. We also studied a nonpolar amino acid and carboxylate functional groups for comparison. Computation of the ideal performance of individual CNTs at atomistic scale is a powerful tool for probing the effect of tip-functionalized CNTs on water and ion transport mechanism. Molecular simulation study suggests that steric effects required for ion rejection compromise fast water conductivity; however, an Asn functional group having polarity and hydrogen bonding capability can be used to balance this trade-off to some extent. To test our hypothesis, we incorporated functionalized CNTs (f-CNTs) into the in situ polymerized selective polyamide (PA) layer of thin film nanocomposite membranes and compared their experimental RO desalination performance. The f-CNTs were found to change the separation environment through modification of cross-linking density, thickness, and hydrophilicity of the PA layer. Asn functionalization led to more cross-linked and thinner PA layer while hydrophilicity is improved compared to other functional groups. Accordingly, water permeance is increased by 25% relative to neat PA with a salt rejection above 98%. Starting from the nanomaterial itself and benefiting from molecular simulation, it is possible to design superior membranes suited for practical applications.

## 1 Introduction

As the gap between water demand and supply widens globally, desalination technologies have become vital. The current capacity of membrane-based reverse osmosis (RO) plants significantly dominates the worldwide desalinated water production,^1^ whose market is estimated to be valued at over US$ 30 billion with an annual growth rate of 55%.^2^ However, operational expenditures and related environmental impacts compromise the sustainability of RO technology.^3^ Accordingly, realizing the ultrapermeable, selective, and robust membranes will assist in addressing these problems.^3−5^ Polyamide (PA)-based thin film composite (TFC) membranes have been prevalently utilized in RO applications for decades.^6^ Nevertheless, their performance is limited by the intrinsic permselectivity of PA, and an upper bound for TFCs has recently been established.^7^ Encouragingly, aquaporins (AQPs) and carbon-based nanomaterials are proposed as the most attractive platforms for designing innovative membranes with ultrahigh water permeability, outperforming the conventional TFCs.^8−11^ Novel strategies that combine the inspiration from nature and the advantages of carbon-based nanomaterials have also emerged recently.

The discovery of AQP channel-forming membrane proteins has elucidated the surprisingly high water/ion selectivity of biological cell membranes.^12,13^ A hourglass-shaped channel narrows down to ∼2.8 Å, imposing a size restriction on ions while allowing the passage of single-file water chains.^14^ Hydrophobic residues, lining the interior part of the channel, mediate the fast water conduction up to a single channel permeability of ∼10^–13^ cm^3^·s^–1^.^14,15^ Meanwhile, size exclusion and electrostatic interactions arising from polar residues prevent the passage of ions, including protons, from reaching a theoretically infinite water/ion selectivity.^16,17^ Understandably, the potential implications of AQPs for desalination have paved the way for biomimetic membranes which incorporate directly either AQPs or AQP-inspired artificial water channels (AWCs).^18−20^ Although AQP-based membranes show vast potential for high-performance desalination, their commercialization is arguably problematic. Transmembrane proteins like AQPs have relatively complex and expensive synthesis routes since they are prone to denaturation.^19−21^ Besides, they necessitate an amphiphilic housing which further complicates the defect-free and large-scale membrane fabrication.^19,20^ Given that most of the AQP-based membranes are tested at low pressures (1–10 bar), their stability at RO conditions remains questionable.^19,21^ These challenges prompt the application of biomimicry to synthetic nanochannels which are feasible for practical applications such as seawater and brackish water desalination.

Among the carbon-based next-generation membrane materials, carbon nanotubes (CNTs) have attracted a great deal of attention as ultrafast water channels.^22−24^ Consisting of hexagonal sp^2^ hybridized carbons, hydrophobic and atomically smooth tube walls are mainly responsible for the analogy between CNTs and AQPs.^25−27^ Particularly, single-walled CNTs (SWCNTs) with a subnanometer aperture conducting water as single-file chains are considered as synthetic counterparts of AQPs and thus promising candidates for constructing AWCs.^28,29^ Molecular dynamics (MD) simulations of water-CNT systems reveal the mechanism of rapid water conduction in SWCNTs,^30,31^ the effect of tube diameter^32,33^ and length^34^ as well as metallicity^35^ at the molecular level. As for desalination, MD simulations predict the critical tube diameter as 8.1 Å for complete exclusion of Na^+^ and Cl^–^ ions.^33^ Since the precise control of CNT diameter and synthesis in monochiral form at large scale are technically challenging, functionalization of CNTs emerges as an effective strategy to tailor selectivity.^23^ Bulky functional molecules such as biotin,^36^ straight-chain alkanes, negatively charged dyes, aliphatic amines,^37^ and zwitterion^38^ as well as small groups (COO^–^, NH_3_^+^, OH, and CONH_2_)^39^ lead to enhanced ion selectivity via a gated transport mechanism.

Compared with freestanding, vertically aligned CNT membranes, CNT/PA thin film nanocomposite (TFN) membranes are studied more extensively for RO applications owing to their scalability advantage.^8,23,24^ Increasing permeability and/or fouling resistance is usually aspired upon incorporation of CNTs into the selective PA layer.^9,10^ Nevertheless, pristine CNTs (p-CNTs) are not compatible with common membrane polymers because of their hydrophobic nature and tendency to agglomerate via π–π stacking.^40^ Therefore, functionalization of CNTs is necessary to improve CNT-PA interactions and to decrease the formation of defects. Most of the CNT/PA TFN studies^10,40^ utilize carboxylated CNTs with the exception of reports on the zwitterion,^38^ amine,^41^ and polyacrylamide^42^ functionalization. These studies show that functional molecules attached to CNTs modify the properties of the PA layer, such as cross-linking degree, hydrophilicity, and roughness.

Recently, the unique shape and chemistry of AQPs inspire the modification of carbon-based membranes for high-performance desalination. In addition to the hydrophobic interior surface analogy, the design of the bioinspired carbon channels employs the hourglass geometry,^43,44^ charged and polar functional groups,^45^ or a functional peptide corresponding to filter region of AQPs.^46^ Herein, we employ the amino acid asparagine (Asn) to functionalize our SWCNTs based on its important contribution to water selectivity of AQPs and we exclude the effect of geometry and the presence of other amino acids in the selective region. Asn residues are located near the constriction region of AQPs, as a part of the evolutionary conserved asparagine-proline-alanine (NPA) motif.^14,17^ Owing to their amide containing side chain, Asn residues form multiple transient hydrogen bonds with water molecules as they are adopting a single-file geometry to fit into the constriction region.^17,47^ Thus, we hypothesize that Asn molecules attached to the CNT entrance favor the conduction of water molecules while improving the sieving properties of CNTs. In addition, the amide-containing side chain and the polar structure of the Asn molecule enhances CNT-PA compatibility when functionalized CNTs (f-CNTs) are incorporated into TFN membranes. In order to support our hypothesis, we also examine the effect of a nonpolar amino acid, 8-aminocaprylic acid (ACA), on the permselectivity of CNTs. Additionally, we compare our amino acid modified CNTs with their unmodified form, which are commercially provided carboxylated CNTs. MD simulations of f-CNTs reveal the desalination potential of functional groups while RO filtration experiments with f-CNT/PA TFNs demonstrate their effect on the membrane structure and performance. Comparing the performance of CNTs as individual desalination platforms and as fillers in nanocomposite membranes, we also aim at understanding the gap between novel concepts and feasible applications.

## 2 Methods

### 2.1 Computational Methods

#### Setup and Equilibration

**Figure 1:**
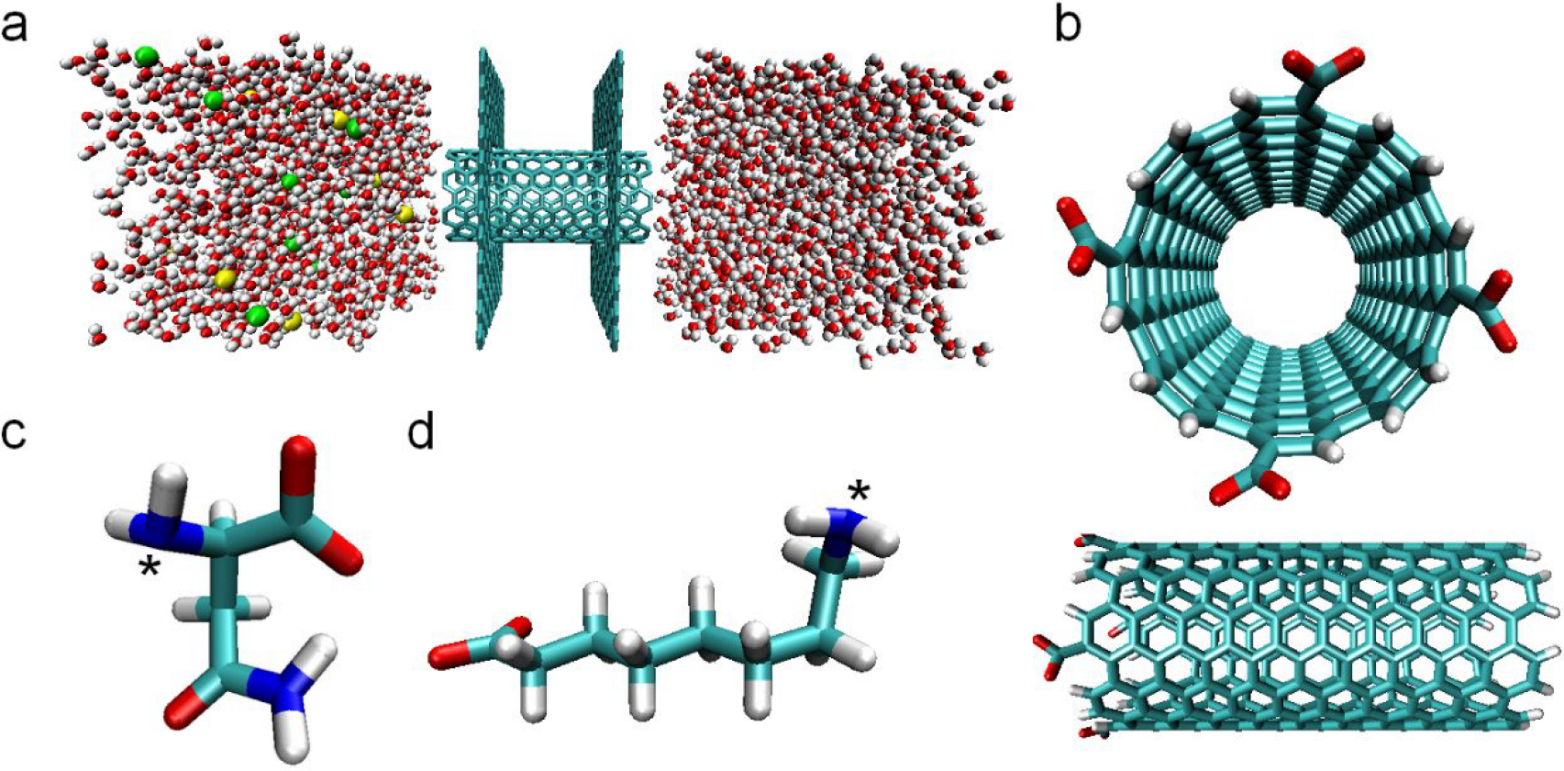
Simulation setup. (a) Side view of the periodic box consisting of saline water layer, CNT (e.g., pristine) surrounded by graphene walls, and pure water layer (from left to right). (b) Top and side views of CNT functionalized with COO^–^ molecules from its tips in tetrads. Structures of (c) Asn and (d) ACA molecules. The asterisk (*) in panels c and d denotes N atoms participating functionalization. (C: cyan, O: red, N: blue, H: white, Na^+^: green, Cl^–^: yellow).

Periodic simulation box (Figure 1a) consists of three layers: (i) a saline water box having an ion concentration of 35 000 ppm, (ii) p- or f-CNT confined by two parallel graphene walls, and (iii) pure water box. The saline water box has dimensions of 30 × 30 × 30 Å and contains 888 water molecules as well as 10 Na^+^ and 10 Cl^–^ ions, while a pure water box having the same dimensions contains 903 water molecules. CNTs are modeled as (8,8) SWCNTs with a diameter of 10.85 Å and a length of 24.6 Å. CNTs are left in their original cylindrical form and not deformed to suit the hourglass geometry of AQPs. Carbon atoms of CNTs and graphene layers are treated as neutral CA-type carbons of AMBER ff94 force field,^48^ and their coordinates are kept fixed throughout the simulations. Geometry optimization and charge calculation of functional groups (Asn, ACA, and COO^–^) are performed by AMBER's antechamber with AM1-BCC charge model.^49^ The chemical structure and charge distribution of molecules are also given (Figure S1). COO^–^ molecules are uniformly bounded to CNT tips in tetrads (Figure 1b). For amino acid functionalization, the backbone nitrogen of Asn or ACA (Figure 1c,d) is bonded to carbon of COO^–^. Systems containing pristine CNT and COO^–^-, Asn-, and ACA-functionalized CNTs will be referred to as PRT, COO, ASN, and ACA, respectively. AMBER ff94 and ff99 force fields^48,50^ are used for parametrization of ions (Na^+^ and Cl^–^) and functional groups, while water molecules are modeled by the modified TIP3P model of Price and Brooks.^51^ Molecular dynamics simulations are performed using the LAMMPS simulation package^52^ and trajectories are visualized by Visual Molecular Dynamics (VMD) v1.9.3.^53^ The van der Waals interactions are modeled by the Lennard-Jones 12–6 potential, and electrostatic interactions are calculated by the particle–particle particle–mesh (PPPM) Ewald summation method, with a 12.0 Å-cutoff for both. The Nose–Hoover thermostat and barostat is used for pressure and temperature control. A time step of 1 fs is used with the SHAKE algorithm. NVT-MD simulations of 100 ps is applied at 1 bar and 30 K for minimization of systems prior to gradual heating with NPT-MD until 298 K in 1 ns. The equilibration is completed after 1 ns of NPT-MD runs at 298 K when constant density is reached.

#### Equilibrium Molecular Dynamics (EMD)

Following the NPT-MD equilibration, graphene walls are removed in order to eliminate their effect on the water density profile and NPT-MD is continued for 6 ns, the last 5 ns of which is used for the analysis of hydrogen bonding, interaction energy, radial distribution function (RDF), and water density profile to obtain potential of mean force (PMF) for water molecules. Details of these calculations are given in Section S2 of Supporting Information.

#### Umbrella Sampling

PMF for Na^+^ and Cl^–^ ions conducted through the CNT is calculated by means of umbrella sampling under EMD conditions. The origin (*r* = 0 Å, *z* = 0 Å) is placed at the center of the CNT so that the entrance and exit are near the *z* = 12 Å and *z* = −12 Å positions, respectively. At the beginning of the biased NPT-MD run performed for sampling, the test ion is placed at *r* = 0 Å, *z* = −22 Å and pulled to *z* = +22 Å along the CNT axis. The collective variable is chosen as the axial component of the displacement of ion. The path is divided into 88 umbrella sampling windows with 0.5 Å width and a harmonic biasing potential in the form of  is applied following the method carried out by Corry.^33^ A force constant of *K*_*r*_ = 0.2 kcal·mol^–1^·Å^–2^ is applied in order to keep the ion near *r* = 0 Å, while a second force constant *K*_*z*_ = 2 kcal·mol^–1^·Å^–2^ is applied to pull the ion to the target position. For each window, a NPT-MD simulation is carried for 600 ps, the first 100 ps of which is for the equilibration and discarded from the calculations. For each system and ion, an NPT-MD run for umbrella sampling is performed for 53.4 ns, reaching a total sampling time of 427 ns. Then, the weighted histogram analysis (WHAM) is performed using the code of Grossfield to construct PMF profiles.^54^

#### Nonequilibrium Molecular Dynamics (NEMD)

Subsequent to NPT-MD equilibration, nonequilibrium NVT-MD simulations with Langevin thermostat are performed to examine water and ion conductivity through the CNTs with graphene walls. Following the approach of Zhu et al.,^26^ a constant external driving force is applied to selected water molecules in order to introduce a hydrostatic pressure difference between the entrance and the exit of CNT. For this purpose, a force constant of 0.05 kcal·mol^–1^·Å^–1^ is applied to water molecules that located within 5-Å-thick layers from the beginning and end of the simulation cell. Resulting pressure difference, Δ*P*, is related to number of molecules in the specified region, *n*, force constant, *f*, and cross-sectional area, *A*, as follows:
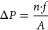
1Applied hydrostatic pressure difference is approximately 140 MPa for all systems. Three independent NEMD simulations of 40 ns are carried out for each system. In addition, NEMD simulations of 10 ns under ∼90, 200, 270, 340, and 700 MPa pressure difference is performed for p-CNT in order to demonstrate the linear relationship between Δ*P* and *f*.

### 2.2 Experimental Methods

#### Materials and Chemicals

Carboxylic SWCNTs with average diameter of 1 nm and length of 1–3 μm (purity >92 wt %) are purchased from Nanografi Co. Ltd. A commercial polysulfone ultrafiltration membrane (Alfa Laval GR40PP) having 100 kDa molecular weight cutoff (MWCO) is utilized as the support layer. The following reagents are used without further purification: MES monohydrate (2-(*N*-morpholino)-ethanesulfonic acid, Sigma-Aldrich, ≥99.5%), EDC (1-ethyl-3-(3-(dimethylamino)propyl) carbodiimide hydrochloride, Thermo Fisher), l-asparagine (Sigma-Aldrich, ≥98%), ACA (8-amino octanoic acid, Sigma-Aldrich, 99%), MPD (*m*-phenylenediamine, Sigma-Aldrich, ≥99%), TMC (1,3,5-benzenetricarbonyl trichloride, Sigma-Aldrich, %98), SDBS (sodium dodecyl benzenesulfonate, Sigma-Aldrich), TEA (triethylamine, Sigma-Aldrich, ≥99.5%), *n*-hexane (Merck, >95%).

#### Amino Acid Functionalization of CNTs

Following the method of Majumder et al.,^37^ EDC is used as cross-linker in order to activate carboxyl groups on CNTs and react with primary amines of amino acids. 0.1 M MES buffer is used to prepare all solutions. First, 50 mg of carboxylated CNT is dissolved in 250 mL of buffer and probe-sonicated in an ice–water bath for 10 min. Then, 25 mL of a 50 mM EDC solution is added to the CNT dispersion and sonicated in ultrasonic bath for 2 h at room temperature. Lastly, 625 μL of a 1 mM amino acid solution is added to the reaction mixture and sonicated for 2 h. The f-CNT dispersion is washed with deionized water and dried in vacuum oven at room temperature. Functionalization of CNTs is confirmed by thermal gravimetric analysis (TGA), X-ray photoelectron spectroscopy (XPS), and Raman spectroscopy. TGA (PerkinElmer Diamond TG/DTA) is carried out with an oxygen feed rate of 100 cm^3^·min^–1^ and a heating rate of 5 °C·min^–1^ to record weight loss until 800 °C to obtain TGA and differential thermogravimetry (DTG) curves. XPS (Thermo Scientific K-Alpha) equipped with an Al Kα radiation source is used to determine the elemental composition. The Raman spectra of f-CNTs are obtained by Raman microscope (Renishaw InVia), focusing a 633 nm laser with a 50× objective lens.

#### Membrane Preparation

CNT/PA TFN membranes are fabricated following the procedure that we previously reported.^55^ In summary, 5 mg of f-CNT is dispersed in 40 mL of deionized water along with 10 mg of SDBS surfactant. Mixture is probe-sonicated for 30 min in ice–water bath and centrifuged at 14 000 rpm for 30 min to recover supernatant. The f-CNT concentration of the supernatant is adjusted to 0.03 mg·mL^–1^ using a UV spectrophotometer. The incorporation and partial alignment of f-CNTs inside of the porous support layer is provided by vacuum filtration. For this purpose, 40 mL of a f-CNT dispersion is filtrated through the support membrane with an area of 40 cm^2^ under a 50 kPa vacuum. Then, interfacial polymerization is carried out to synthesize the PA layer. The support membrane is soaked in the aqueous phase containing 2% (w/v) MPD, 2% (w/v) TEA, and 0.04% (w/v) SDBS for 20 min, and excess solution is removed by means of a glass roller. Next, the membrane surface is brought into contact with the organic phase containing 0.5% (w/v) TMC dissolved in hexane for 90 s. The resulting TFN membrane is heat-treated in an oven at 68 °C for 10 min. For the synthesis of TFC membranes, the same procedure is performed utilizing a support layer that is not incorporated with any f-CNTs. At least three replicas for each TFC/TFN membrane are prepared under identical synthesis conditions.

#### Membrane Characterization

Morphology of membranes is evaluated using field emission scanning electron microscope (FE-SEM, Zeiss Ultra Plus) and atomic force microscope (AFM, Bruker Dimension Icon). Membrane areas of 10 × 10 μm^2^ are scanned in tapping mode to examine surface roughness at nanoscale. Membrane samples are analyzed by Fourier transform infrared spectroscopy (FTIR, PerkinElmer Spectrum One) to verify formation of PA layer. XPS analysis is also carried out for membrane samples as described for CNTs. The cross-linking density of PA is determined on the basis of the surface elemental composition. Intercalation of CNTs within the PA chains is examined by X-ray diffraction (XRD, Rigaku Smartlab) with Cu Kα radiation. To investigate surface hydrophilicity, water contact angle is measured by sessile drop method by means of a tensiometer (KSV Attension Theta). Mechanical strength of membranes is characterized by dynamic mechanical analysis (DMA, PerkinElmer Diamond) via tension mode at heating rate of 3 °C·min^–1^ and frequency of 1 Hz. Lastly, separation performance is evaluated using a cross-flow filtration system (GE Osmonics Sepa CF II). A 2 000 ppm aqueous NaCl solution (*T* = 25–27 °C and pH = 6–6.5) is used as feed. Membranes are compacted at 16 bar until permeate flux reaches steady state. Then, the permeate is collected under transmembrane pressure of 15.5 bar, which is common for testing of laboratory-scale RO membranes. The conductivity of the feed and permeate samples are measured by a conductometer (inoLab Cond 7110, WTW) to determine NaCl rejection rate (%).

## 3 Results and Discussion

### 3.1 Simulated Desalination Performance of Individual CNTs

EMD simulations are performed for four different systems (namely PRT, COO, ASN, and ACA) to understand how functional groups interact with water molecules and ions. Our simulation setup consists of a p- or f-CNT confined by two parallel graphene walls placed in the middle of the saline and pure water layers (Figure 1a). Bounded to CNT tips in tetrads (Figure 1b–d, Figure S1), functional groups control the entry of molecules through steric and electrostatic interactions. Moreover, their effect extends throughout the channel as they modify interactions among the water molecules. For all systems, water molecules confined in the CNTs adopt a five-molecule chain geometry (Figure S2), similar to previous reports on (8,8) SWCNTs.^35^ In addition, the time-averaged number of confined water molecules within the CNTs is ∼40 for all systems (Figure S3a).

**Figure 2:**
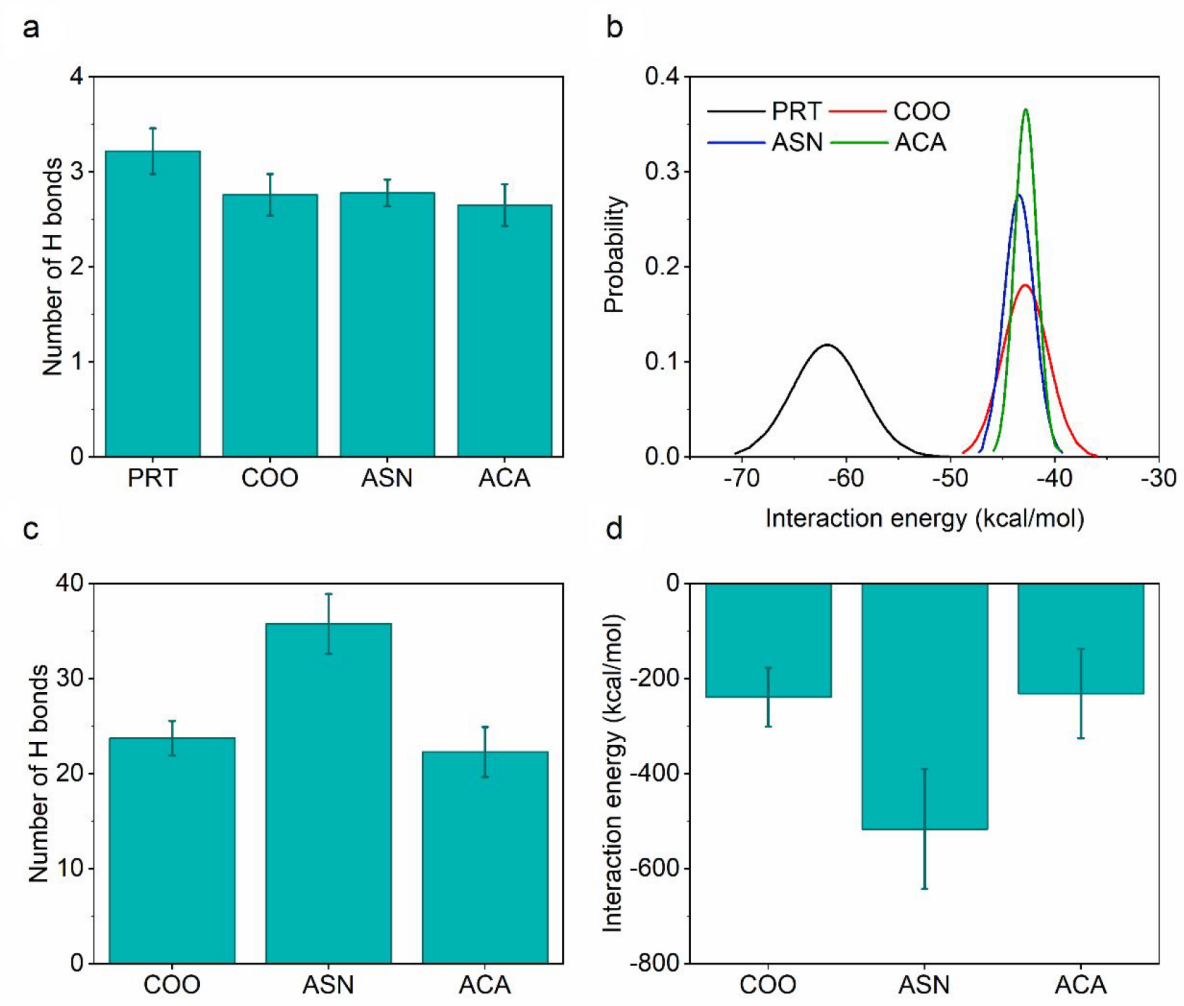
Interaction strength between water–water and water–functional group. (a) Time-averaged number of per-molecule hydrogen bonds formed among water molecules confined in CNTs. (b) Probability distribution of per-molecule interaction energy of confined water molecules (averaged over different configurations with 1 ns interval). (c) Time-averaged number of total hydrogen bonds formed between water molecules and functional groups. (d) Time-averaged energy of interactions formed between water molecules and functional groups.

However, in spite of the similar confinement geometry and approximate number of confined water molecules, the number of hydrogen bonds formed among the confined waters is reduced in f-CNTs when compared with PRT (Figure 2a). The trend in hydrogen bonds is supported by per molecule interaction energy of water molecules confined in CNTs (Figure S3b) with a lower energy for f-CNTs than that of PRT. Therefore, with functionalization of the CNT, the environment of water molecules is altered, which decreases the CNT–water interaction energies by approximately 21% in f-CNTs compared to PRT. On the other hand, although per molecule interaction energy of water molecules confined in f-CNTs is comparable with each other, its probability distribution varies by the functional groups (Figure 2b). As the steric hindrance at the entrance increases, number of allowable different configurations to be adopted by water molecules decreases. Hence, water molecules confined in ACA are conducted as a more ordered chain, showing a narrow probability distribution. For ASN, COO, and PRT, the distributions gradually broaden while entrance effects become less prominent. In these distributions, molecules deviating from the mean energy appear in the left-hand side (high-energy states, more favorable) or right-hand side (low-energy states, less favorable) of the distribution. When confined water–water interactions within the tube become less favorable, the probability of finding a molecule in the right-hand side increases. From this point of view, the right-hand side of the distribution for COO, falling outside the overlapping ASN and ACA distributions, indicates that some water molecules in this system have less favorable interactions with other waters. Similarly, when the left-hand side of distribution for ASN and ACA is compared, ASN seems to have the most energetically favorable water–water interactions.

The number of hydrogen bonds formed by functional groups with all water molecules in the system throughout EMD simulations and the energy of their interaction (Figure 2c,d) follow the same trend. The correlation between the number of hydrogen bonds and the total interaction energy also implies the energetic dominance of electrostatic effects. Because of the nitrogen and oxygen atoms in the amide-containing side chain, ASN forms the most favorable water interactions. On the other hand, the interaction energy and number of hydrogen bonds are comparable for COO and ACA, albeit the latter has a larger number of atoms. The weaker water interaction of ACA is attributed to the nonpolar aliphatic chain of the functional group. The RDFs evaluated for carboxylate oxygens of COO^–^, Asn, and ACA molecules and water oxygens exhibit a similar intermolecular O–O distance and peak intensity (Figure S4a–c). The RDFs also characterize the hydration shells around the amide oxygen and nitrogen of Asn side chain (Figure S4d,e), demonstrating that the favorable water interactions of Asn mainly arises from its side chain. Furthermore, the distribution of water molecules within the simulation cells are depicted via 2D atomic density maps (Figure S5). Hydrophobic and atomically smooth graphitic walls of nanotubes promote alignment of water molecules both at the entrance and along the tube axis in the PRT system. However, functional groups introduce a nonuniform water distribution at the tube entrance. Particularly, steric hindrance introduced by the ACA group induces relatively wide water-poor regions at the tube entrance. Since 2D-density maps are generated on the basis of the density values averaged over the EMD trajectories, they also suggest that the mobility of functional groups differs (Figure S5). ACA molecules tend to have a limited number of configurations, while Asn molecules are more flexible and do not define certain sterically inhibited regions that exclude water molecules.

**Figure 3:**
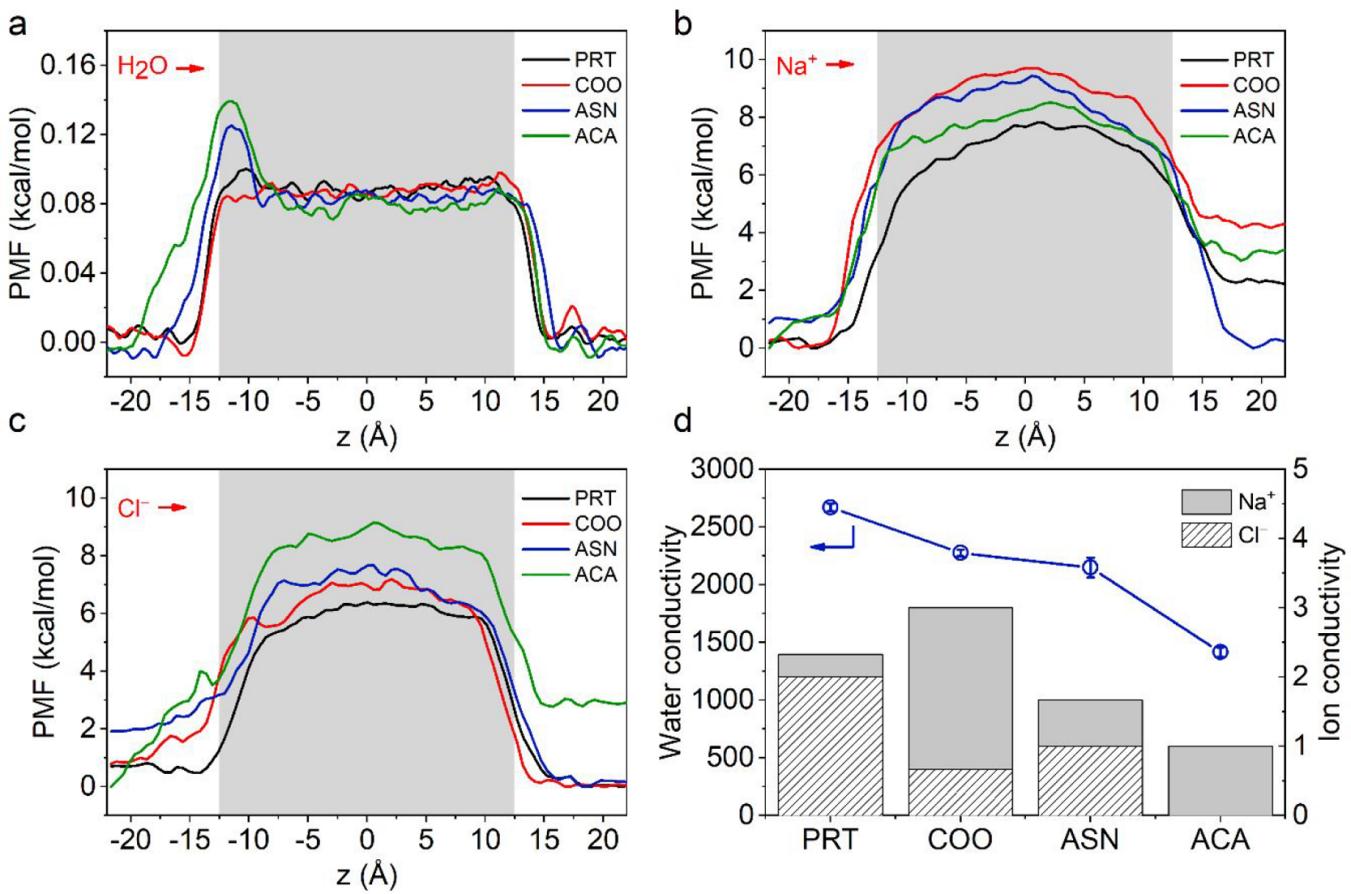
Energy barriers along the CNT axis and conductance of water and ions. (a) PMF for water molecules. (b) PMF for Na^+^ ion. (c) PMF for Cl^–^ ion. (Shaded areas in panels a–c indicate the entrance and exit of CNTs with center at *z* = 0 Å.) (d) Number of water molecules and ions conducted through CNTs obtained from NEMD simulations. (The average number of molecules calculated from three independent 40 ns-long simulations is reported for each system. Raw data and associated standard deviation values are given in Table S2.)

Free energy profiles of water and ions at the entrance of the CNTs observed from potential of mean force (PMF) calculations allow us to characterize the energy barrier encountered by water molecules and ions entering the CNTs, which significantly affects the water and ion transport. The free energy profile for water (Figure 3a) derived from the density profile in the EMD simulations suggests that water molecules need to overcome only a small energy penalty (less than 0.15 kcal·mol^–1^) for all systems upon entry. Compared with PRT, the COO system does not exhibit any additional barrier because of its small molecular size. The relatively higher energy barriers in ASN and ACA systems are attributed to steric hindrance due to the bulkier functional groups. Because of the low atomic density of ions, unbiased EMD simulations do not provide statistically reliable information about interactions between functional groups and ions. Therefore, energy barriers for ion transport are evaluated on the basis of a biasing approach that is the umbrella sampling method in which a test ion is pulled along the CNT axis by applying a harmonic potential. For uncharged nanochannels, the main reason for energy penalty of ion conduction is that ions lose the contact with coordinated water molecules in their hydration shell to fit narrow confined spaces. Comparing the RDF of an ion in bulk phase to that of the test ion conducted within PRT (Figure S6), we show that second hydration shells of both Na^+^ and Cl^–^ ions are deformed, implying the hindered water–ion interactions and governing the energy barriers in PRT (Figure 3b,c). As for f-CNTs, the energy barrier is contributed by the further narrowing effect at the entrance as well as the electrostatic repulsive and attractive interactions formed between functional groups and ions. COO system exhibits a lower energy barrier for Na^+^ at the entrance (until −15 Å) because of the small size and negatively charged oxygens of the functional group; however, the barrier rises sharply by exceeding the PRT in line with the literature^39^ since breaking the attractive interactions between the ion and oxygens during the ion diffusion is penalized. COO is followed by ASN and ACA, which show a slightly higher barrier at the entrance but lower penalty during the diffusion because of the charge distribution on these functional groups. For Cl^–^ ion, negative charges on the COO system provides a higher barrier at the entrance compared to PRT. However, the highest energetic penalty for Cl^–^ conduction is observed for the bulky ACA group with the effective size exclusion ability. This can be attributed to the greater size and coordination number of the Cl^–^ ion,^56^ which makes the steric effects dominant in its rejection. Both Na^+^ and Cl^–^ do not encounter with any functional group while entering PRT; therefore, compared with f-CNTs, PRT has the lowest energy barriers at the entrance and throughout the ion diffusion inside the tube. Interestingly, PRT system shows a lower energy barrier for Cl^–^ than Na^+^ both at the entrance of CNTs and during the transport through CNT, implying that carbon atoms of CNT favors the transport of Cl^–^ ions more than Na^+^ ions due to the greater Lennard-Jones potential depth (Table S1). We note that when the channel is neutral and wide enough to accommodate both ions, van der Waals interactions may determine the ion selectivity.

Regarded as the computational counterpart of filtration experiments, NEMD simulations provide the water and ion conduction of CNTs under a hydrostatic pressure difference. In these simulations, water molecules and ions move toward the channel in a chaotic manner and compete for entrance; thus, the attractive interactions between functional groups and diffusing molecules become effective at the entrance (see the supplementary animated video and Section S15 in Supporting Information). These interactions play an important role in governing the water/ion or ion/ion selectivity of channels. Furthermore, the influence of functional groups on the hydrogen bonding and arrangement of water molecules also modify water conductivity. Water and ion conductivity of the studied channels are obtained via NEMD simulations (Figure 3d). When f-CNTs are compared with PRT, functional molecules inevitably decrease water permeability. In the COO system, both water permeability and water/Na^+^ selectivity are compromised because of favorable electrostatic interactions, low energy barrier at the entrance, and high conductivity shown for Na^+^ ion compared with PRT. On the contrary, Cl^–^ conductivity, which is notably high in PRT because of a low energy barrier, is effectively decreased in COO. However, total ion conductivity declines in the ASN system while retaining the water permeability compared with the COO system. This is attributed to several advantages of the Asn group: (i) charge distribution on the molecule preventing a preferential ion transport (Figure S1); (ii) hydrogen donors and acceptors in the Asn side chain which favors the water intake (Figure 2c,d, Figure S4d,e) and increases water/ion selectivity of the channel; and (iii) narrow interaction energy distribution (Figure 2a) and ordered configuration of water molecules, which further accelerates the water diffusion. Lastly, the ACA system shows the lowest total ion conductivity with 100% rejection of Cl^–^ during a 40 ns simulation time for three independent runs along with the lowest water permeability, corresponding to high energy barriers arising from large and nonpolar structure of the functional molecule. Compared with PRT, ASN and ACA systems sacrifice ∼19% and ∼47% of the water flux in return for a decrease of ∼28% and ∼57% in total ion flux, respectively. For the sake of a permeability-selectivity trade-off, our computationally optimized CNT design employs Asn as the functional group; nevertheless, the ACA group may also be utilized where particularly high ion rejection is required.

**Figure 4:**
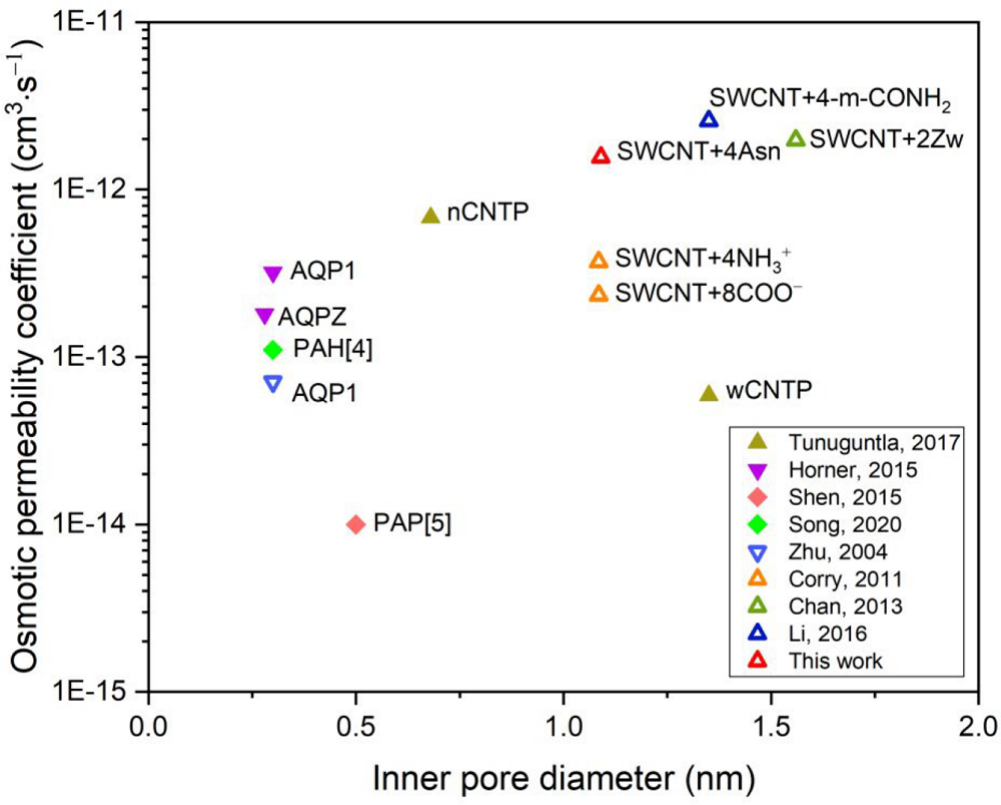
Osmotic water permeability of single channels (namely AQPs, CNTs, and AWCs) against the inner pore diameter. Note that the inner diameter of their pristine form is given for functionalized CNTs. Both experimental (solid symbol) and molecular simulation (open symbol) studies are included. Upward triangle, downward triangle, and diamond symbols indicate categories of CNT, AQP, and AWC, respectively. Details are available in Table S3.

One of the important considerations in the NEMD simulations is the high pressure difference being a few orders of magnitude higher than the experimental transmembrane pressure.^35,38,39,45^ Introducing such high pressures ensures that statistically meaningful data is obtained within reasonable simulation times.^57^ On the basis of the linear relationship between the pressure difference and solvent flux, the number of conducted solvent molecules can be extrapolated to determine the flux at experimentally applicable pressures or the osmotic permeability coefficient of the channels. We performed additional NEMD simulations for PRT system under ∼90, 200, 270, 340, and 700 MPa pressure difference to show the linear relationship is preserved until 340 MPa (Figure S7). Therefore, the number of water molecules conducted under 140 MPa can be used to predict the osmotic permeability coefficient of our channels (see Section S9 in Supporting Information). Accordingly, we compared the water permeability of several water channels studied experimentally or computationally (Figure 4). Designed to improve efficient ion rejection (∼100%), f-CNTs have comparable water permeability to that of experimentally studied AQPs, AWCs, and CNT porins (CNTP). For the same initial pore diameter (∼1.1 nm), 4Asn functionalization resulted in higher water permeability compared with other highly selective 8COO^–^ and 4NH_3_^+^ functionalized CNTs. It also has comparable permeability to wider CNTs employing different modification strategies such as bulkier functional groups or inner wall functionalization to improve selectivity. Among these several f-CNTs, our ASN channel shows potential with its tailored water/ion selectivity. It is also demonstrated that predicting the water permeabilities within one order of magnitude of experimentally measured values, MD simulations have proved to be useful in designing water channels (Figure 4).

### 3.2 Experimental Desalination Performance of CNT/PA TFN Membranes

#### 3.2.1 Characterization of f-CNTs

**Figure 5:**
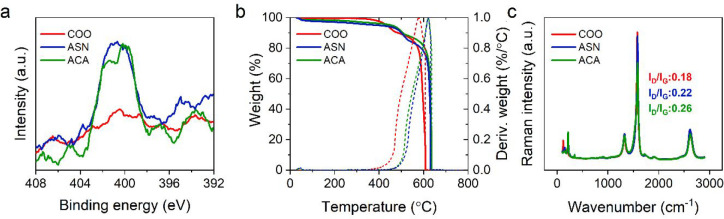
Characterization of f-CNTs. COO is commercially provided and modified via carbodiimide mediated coupling to obtain ASN and ACA. (a) High-resolution N(1s) region of XPS survey spectra. (b) TGA and DTG curves. (c) Raman spectra showing G band (∼1 600 cm^–1^) and D band (∼1 300 cm^–1^).

Prior to CNT/PA TFN membrane synthesis, commercially provided carboxylated CNTs (COO) were modified with Asn and ACA molecules via carbodiimide mediated coupling between the carboxyl groups of CNTs and amine groups of amino acids.^37^ Amino acid functionalized CNTs will be referred to as ASN and ACA henceforth. Asn and ACA-functionalization was confirmed via XPS demonstrating the presence of nitrogen atom in their high-resolution N(1s) spectra (Figure 5a). The survey spectra (Figure S8) and atomic composition of f-CNTs (Table S4) obtained by XPS analysis also support the functionalization. In addition, TGA curves of ASN and ACA are compared to COO to examine the effect of functionalization on the thermostability (Figure 5b). Weight losses around 100 °C, 200–400 °C, and 400–600 °C, are related to the vaporization of solvents, decomposition of amorphous carbon, and decomposition of functional groups, respectively. Decomposition of sp^2^ hybridized carbon atoms belonging to SWCNTs takes place around 600 °C, as reported in the literature.^58^ TGA and DTG curves (Figure 5b) indicate that both Asn and ACA functionalization shifts the decomposition to slightly higher temperatures compared with COO. Since functionalization methods involving acidic treatment or rigorous sonication may introduce additional defects in the CNTs, we examine the structural stability of our f-CNTs via Raman spectroscopy. Tangential Raman modes of CNTs at 1 600 cm^–1^ are referred to as the G band, while the second peak at 1 300 cm^–1^ is called the D band, which is associated with the defects; thus, the concentration of defects can be quantitatively compared on the basis of the *I*_D_/*I*_G_ ratio.^59,60^ Accordingly, our functionalization method slightly deforms the CNTs as indicated by the increase in the *I*_D_/*I*_G_ ratio (Figure 5c). Nonetheless, the sp^2^ hybridization and tube structure of f-CNTs are still preserved, and their *I*_D_/*I*_G_ ratio is similar to reports on other f-CNTs.^59^

#### 3.2.2 Characterization of CNT/PA TFN Membranes

In our previous study,^55^ we employed vacuum-filtration to partially align CNTs within the pores of the support layer prior to the formation of the selective PA layer. Important parameters such as pore size of the support, CNT loading, and interfacial polymerization conditions such as monomer concentration and reaction time were optimized in order to maximize vertical alignment of the CNTs and separation performance. Therefore, TFN membranes in this study are synthesized using the developed method, changing only the functional groups on the CNTs. Synthesized membranes will be referred to as M-TFC, M-COO, M-ASN, and M-ACA henceforth. FTIR spectra of TFC and TFN membranes demonstrate that characteristic absorption bands of amide bonds in PA structure^61^ appear, confirming the formation of a selective PA layer via interfacial polymerization (Figure S9). Furthermore, the cross-linking density of the selective layer is evaluated on the basis of the atomic composition of PA obtained by XPS analysis (Table 1, Figure S10). Considering the interfacial polymerization between MPD and TMC monomers, O/N ratio of resulting PA layer varies between 1 (theoretically fully cross-linked) and 2 (theoretically fully linear) depending on the degree of cross-linking.^62^ High cross-linking density is desirable since it leads to a thinner PA layer having both high NaCl rejection and water permeance.^63^ Therefore, we also correlated the O/N ratio with PA thickness. With an O/N ratio of 0.95, TFC has the highest cross-linking density and thinnest PA. However, the incorporation of CNTs disrupts the cross-linking because of intercalation of nanotubes within the PA chains while thickening the PA layer. By modifying the interactions between the CNTs and PA, functional groups affect the degree of this disruption. The Asn group has the most favorable intermolecular interactions with PA because of its side groups containing nitrogen and oxygen atoms. In addition, the flexibility and mobility of this functional molecule minimize the sterically inhibited regions and allow PA to surround the CNTs, similar to water molecules (Figure S5). Correspondingly, the cross-linking density of M-ASN is higher than that of other TFNs. This also results in a notably thinner PA layer compared with M-COO and M-ACA. In addition, poor cross-linking of M-ACA is attributed to the bulk and constraint structure of the ACA group (verified by simulation, Figure S5), which weakly interacts with polymer and hinders the free movement and cross-linking of PA chains during the polymerization reaction. Intercalation of f-CNTs within the PA chains is also examined by XRD analysis (Figure S11). Although the sensitivity of XRD does not allow comparison of all membranes in detail, the slight shift of the peak at 2θ = 18.3° in M-ACA implies a greater chain intercalation in this membrane.

**Table 1:** Cross-Linking Density and PA Thickness of TFC and TFN Membranes Embedded with f-CNTs^a^

	atomic concentration				
membrane label	O(1s)	N(1s)	C(1s)	O/N ratio	PA thickness (nm)
M-TFC	11.6	12.2	76.3	0.95	193
M-COO	18.1	10.8	71.1	1.68	381
M-ASN	20.2	14.4	65.4	1.40	225
M-ACA	24.8	13.4	61.8	1.85	484

a Data belonging to M-TFC and M-COO are taken from our previous work.^55^

**Figure 6:**
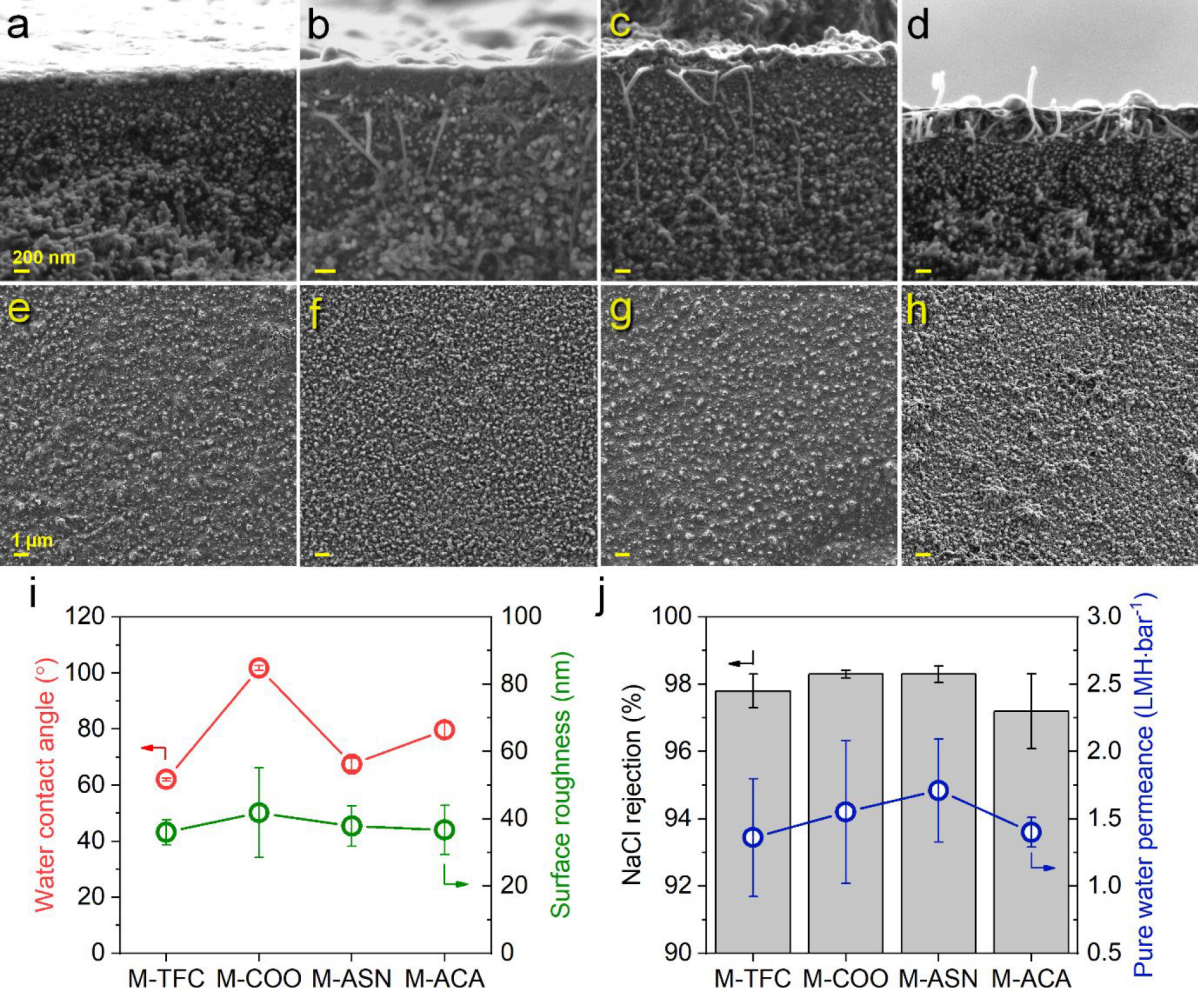
Morphology and separation performance of TFC and TFN membranes. Cross-sectional SEM images of (a) M-TFC, (b) M-COO, (c) M-ASN, and (d) M-ACA. Surface SEM images of (e) M-TFC, (f) M-COO, (g) M-ASN, (h) M-ACA. (i) Water contact angle and surface roughness of synthesized membranes. (j) NaCl rejection and pure water permeance of synthesized membranes. Scale bars in cross-sectional and surface SEM images correspond to 200 nm and 1 μm, respectively. Error bars in water contact angle and surface roughness data represent the standard deviation of measurements taken from three different regions of the same membrane coupon. Error bars in NaCl rejection and pure water permeance represent standard deviation of three independent membrane replicas prepared under identical conditions. Raw data are given in Table S5.

The cross-section and surface of membranes are imaged via FE-SEM to reveal morphological changes undergone upon CNT incorporation. Cross-sectional images (Figure 6a–d) demonstrate deep penetration and partial vertical alignment of CNT bundles under the influence of vacuum filtration. Also, the ends of CNT bundles seem to be extending throughout the selective PA layer (Figure S12). CNTs being partially aligned and embedded in the top selective layer is useful for exploiting the effect of functional groups on water and ion transport. Surface SEM images (Figure 6e–h) demonstrate a rough ridge-and-valley structure of PA in all synthesized membranes. The PA layer in M-COO and M-ASN seems mostly uniform similar to M-TFC while aggregates are visible in M-ACA. Probably, the surface uniformity of M-ACA is disrupted since PA cannot seamlessly surround ACA due to their poor interaction with the polymer. The surfaces of synthesized membranes are also imaged via atomic force microscopy (AFM) (Figure S13), which further demonstrates the nonuniform surface of M-ACA. In addition, the surface roughness of the membranes is determined from AFM analysis (Figure 6i). The intrinsic roughness of PA dominates the overall roughness of the synthesized membranes; however, a slight increase in surface roughness upon CNT addition is noted. Furthermore, the hydrophilicity of TFC and TFN membranes is evaluated via water contact angle measurement (Figure 6i). Our neat PA in M-TFC is essentially hydrophilic having water contact angle of approximately 60°; however, COO significantly decrease wettability in M-COO with water contact angle of ∼100°. Apparently, small carboxyl groups cannot effectively compensate the hydrophobic nature of CNTs, albeit their polar structure. Hydrophilicity of M-ACA lies between M-TFC and M-COO, while Asn notably increases wettability. M-ASN has comparable water contact angle to that of neat PA, almost completely compensating the intrinsic hydrophobicity of CNTs. This is attributed to additional hydrogen-bonding sites on this functional group contributing to the overall hydrophilicity of the resulting membrane. Lastly, we evaluated the mechanical strength of our membranes via DMA analysis (Figure S14). As previously reported,^55^ storage moduli (E′) increases upon CNT addition because of the high mechanical robustness of CNTs, resulting in higher E′ in all TFN membranes compared with TFC. For TFN membranes incorporating the same CNT loading, E′ and mechanical strength is correlated with the PA layer thickness, following the trend of M-ACA > M-COO > M-ASN.

#### 3.2.3 Separation Performance of CNT/PA TFN Membranes

NaCl rejection and pure water permeance of synthesized membranes are evaluated via cross-flow RO filtration tests (Figure 6j). M-ACA has lower NaCl rejection and slightly higher water permeance compared with M-TFC. On the basis of our simulation data, the bulky and comparably rigid ACA group, which introduces effective steric hindrance at the CNT entrance, seems to provide the best ion rejection performance. Nevertheless, the interaction between the functional group and PA has the dominant impact on the membrane selectivity when f-CNTs are embedded in TFN structure. The bulky and nonpolar ACA molecules weakly interact with the polymer matrix, resulting in a poorly cross-linked, thick, and nonuniform PA (Table 1), with possible pinhole defects (Figure 6h). Besides, the water permeance is lower than TFNs embedding other f-CNTs, in parallel with the simulations where the highest energy barrier for water transport is observed for ACA (Figure 3a). Despite its lower cross-linking density, thicker selective layer, and hydrophobic surface, M-COO has higher water permeance and NaCl rejection compared with M-TFC. Considering the fast water conductivity of COO in our simulations, we predict that in-channel water transport contributes to both water permeance and water/ion selectivity in M-COO. As we observe in M-ACA, the properties of the PA layer have a dominating effect on the selectivity of TFNs; thus, high ion conductivity of COO found in simulations does not reduce NaCl rejection of M-COO. However, M-ASN has the highest water permeance with retained NaCl rejection. Verifying our hypothesis, the high hydrogen bonding capacity of the Asn group (supported by simulation in Figure 2c) improves hydrophilicity and further promotes in-channel water transport of f-CNTs. Moreover, favorable interactions between Asn and PA facilitate the formation of a uniform, thin, and highly cross-linked selective layer. As a result, Asn increases the permselectivity through its combined effect on water selectivity of CNTs and surrounding PA matrix. Collectively, compared with M-TFC, M-COO and M-ASN systems gain ∼14 and ∼25% of the water permeance respectively along with a slight increase in NaCl rejection. It should be noted that Figure 6j indicates a considerable standard deviation for water permeance of the replicate membranes including M-TFC (see also Table S5). We attribute this variation mainly to the nonuniform pore size distribution of the commercial ultrafiltration membrane used as the support layer as discussed in our previous work.^55^ The standard deviation for water permeance data in Figure 6j varies between 0.11–0.53 LMH · bar^–1^. This range is similar to variations (0.4–0.55 LMH · bar^–1^) reported in the literature.^41,64−66^ Finally, it is worthwhile to mention that the long-term operational stability is important for practical applications of developed membranes. In our previous work employing the same membrane fabrication method, we demonstrated high stability of CNTs within the TFN membranes under dynamic and static test conditions.^55^

Lastly, we compare the desalination performance of our M-ASN membrane (ASN/PA) with other AQP/PA and f-CNT/PA membranes synthesized via in situ interfacial polymerization (Table 2). Cross-comparison of different reports on TFN membranes is problematic because of several factors. First of all, pinhole defects within the selective PA matrix and small voids at the filler-PA interface may considerably increase water permeance at the expense of a moderate reduction in the salt rejection. Furthermore, the utilized support layer and thickness of the PA layer may present additional resistance to water transport. Testing conditions such as transmembrane pressure and feed concentration further complicate the performance evaluation. To partially exclude these effects, we base our comparison on permselectivity of TFN membranes rather than permeance and/or salt rejection (see Section S13 in Supporting Information for calculation details). We also specify the improvement in permselectivity relative to TFC as an indicator of the contribution of the filler itself in the achieved permselectivity. Accordingly, permselectivity of AQP/PA and f-CNT/PA TFN membranes presented in Table 2 vary between ∼2.0 and ∼7.0. Despite the significant improvement compared with their TFCs, f-CNT/PA membranes^38,41^ show permselectivity values of 2.0–2.5, falling behind the AQP/PA membranes except the report on polyacrylamide-functionalized large-diameter multiwalled CNTs.^42^ It is also worthwhile to mention that large-diameter CNTs^41,42^ and using high loads of CNTs^38^ contribute to the high permselectivity improvement in f-CNT/PA studies. However, AQP/PA membranes^64−67^ provide 0–20% improvement with permselectivities within 3.0–6.92. With permselectivity of 3.7 and improvement of 30%, our AQP-inspired functionalization strategy outperforms most of the AQP/PA and f-CNT/PA membranes, ultimately showing that permselectivity can be effectively tailored via simulation-assisted design of filler materials.

**Table 2:** Comparison of Our Work with Several AQP/PA and CNT/PA Membranes Studied in the Literature^a^

filler	testing conditions^b^	water flux^c^, LMH	salt rejection, %	perm-selectivity (A/B)	improvement in perm-selectivity, %	ref
Asn func. SWCNTs, D^d^: 1 nm	15.5 bar, 2 000 ppm	26.5	98.3	3.73	30.1	this work
zwitterion func. SWCNTs, D: 1.5 nm	36.5 bar, 2 500 ppm	48.5	98.6	2.05	73.3	(38)
amine func. MWCNTs, D: 5–20 nm	15 bar, 2 000 ppm	56.0	97.3	2.40	89.7	(41)
polyacrylamide func. MWCNTs, D: 20–30 nm	15.5 bar, 2 000 ppm	48.4	98.9	6.50	93.1	(42)
AQPZ containing proteoliposomes	5 bar, 584 ppm	18.1	96.9	6.92	20.2	(64)
AQP containing proteoliposomes	10 bar, 584 ppm	39.2	97.1	3.52	21.5	(65)
AqpZ-containing polymersomes	5 bar, 500 ppm	29.2	93.5	3.12	13.4	(66)
AQP-containing DOPC proteoliposomes	55 bar, 32 000 ppm	21.0	99.0	3.52	0.00	(67)

a Improvement in permselectivity is calculated relative to performance of TFC membrane reported in the related study. Details are given in Table S6.

b Hydraulic pressure difference and NaCl concentration of the feed solution.

c Pure or salt water flux is reported. Note that this is considered in the calculation of permeability coefficients.

d The abbreviation D stands for diameter.

## 4 Conclusion

AQP-inspired modification of carbon-based nanomaterials presents remarkable advantages such as scalability and robustness. In this study, we combined molecular simulations and experiments to design amino acid functionalized CNTs having enhanced permselectivities. We hypothesized that Asn amino acid, located near the constriction region of AQPs and contributing to fast water conduction, can improve water/ion selectivity of CNTs when utilized as a functional group. We also examined the desalination performance of COO^–^- and ACA-functionalized CNTs to support our hypothesis. MD simulations demonstrated that the trade-off between water permeability and ion rejection observed for COO^–^ and ACA can be overcome by Asn because of its polarity and high hydrogen bonding capability. When incorporated into the PA layer of TFN membranes, Asn promotes the formation of a thin, highly cross-linked, and notably hydrophilic selective layer through its favorable interactions with the polymeric matrix. Demonstrated by RO filtration experiments, Asn modification improves water permeance by 25% compared with the TFC membrane while retaining the salt rejection above 98%. Consequently, Asn functionalization advances the desalination performance by modifying CNTs in two different ways: increasing the water selectivity of the channel itself and improving its interactions with the surrounding polymeric matrix. Therefore, both simulations and experiments support our initial hypothesis suggesting that permselectivity of CNTs and embedding membranes can be tailored via AQP-inspired functionalization strategies. Our approach can be extended to include other key features of AQPs such as hourglass geometry or charged amino acids within the selectivity filter. Overall, our work demonstrates that bridging the atomistic scale investigation of the material with actual membrane performance is an efficient strategy to reduce the gap between innovative material platforms and practical membrane applications.

## Supporting Information Available

Additional details of molecular dynamics simulations and analysis of trajectories including radial distribution functions and 2D water density maps; additional characterization details of TFC and TFN membranes including FTIR, XPS, and XRD spectra as well as a description of the animated video (PDF)

## Notes

Animated video showing the permeation of water molecules and ions through Asn-functionalized CNT.
