# Supplementary material for: Desalination Potential of Aquaporin-Inspired Functionalization of Carbon Nanotubes: Bridging Between Simulation and Experiment
Source: ACS Appl Mater Interfaces. 2022 Jun 8;14(24):28174–85. doi: 10.1021/acsami.2c03700 (PMC9227712; doi:10.1021/acsami.2c03700)
Supplement: Supplementary file 1 — am2c03700_si_001.pdf [file am2c03700_si_001.pdf]

SUPPORTING INFORMATION

Desalination Potential of Aquaporin-Inspired Functionalization of Carbon Nanotubes: Bridging Between Simulation and Experiment

Aysa Güvensoy-Morkoyun,[a] Sadiye Velioğlu,[a,b,*] M. Göktuğ Ahunbay,[a] and Ş. Birgül Tantekin-Ersolmaz[a,*]

[a] Istanbul Technical University, Department of Chemical Engineering, Maslak, Istanbul, 34469, Turkey

[b] Institute of Nanotechnology, Gebze Technical University, Kocaeli, 41400, Turkey

* Corresponding authors: ersolmaz@itu.edu.tr; sadiyevelioglu@gtu.edu.tr

S1. Chemical structure and charge distribution of functional groups..3
S2. Details of trajectory analysis 4
S3. Analysis of water molecules confined in CNTs 5
S4. Radial distribution functions...6
S5. 2D water density maps..7
S6. Ion-water and ion-CNT interactions..8
S7. Ion and water conductivities estimated from NEMD simulations.......................................9
S8. Relation between water flux and hydrostatic pressure difference in PRT.........................10
S9. Comparison of single channel water permeabilities..11
S10. Characterization of f-CNTs via XPS 12
S11. Characterization of TFC and TFN membranes 13
S12. Separation performance of TFC and TFN membranes...17
S13. Equations for the calculation of water and salt permeability coefficients 18
S14. Comparison of experimental performance with the literature...19
S15. Description of the animated video 20
References..21

S1. Chemical structure and charge distribution of functional groups

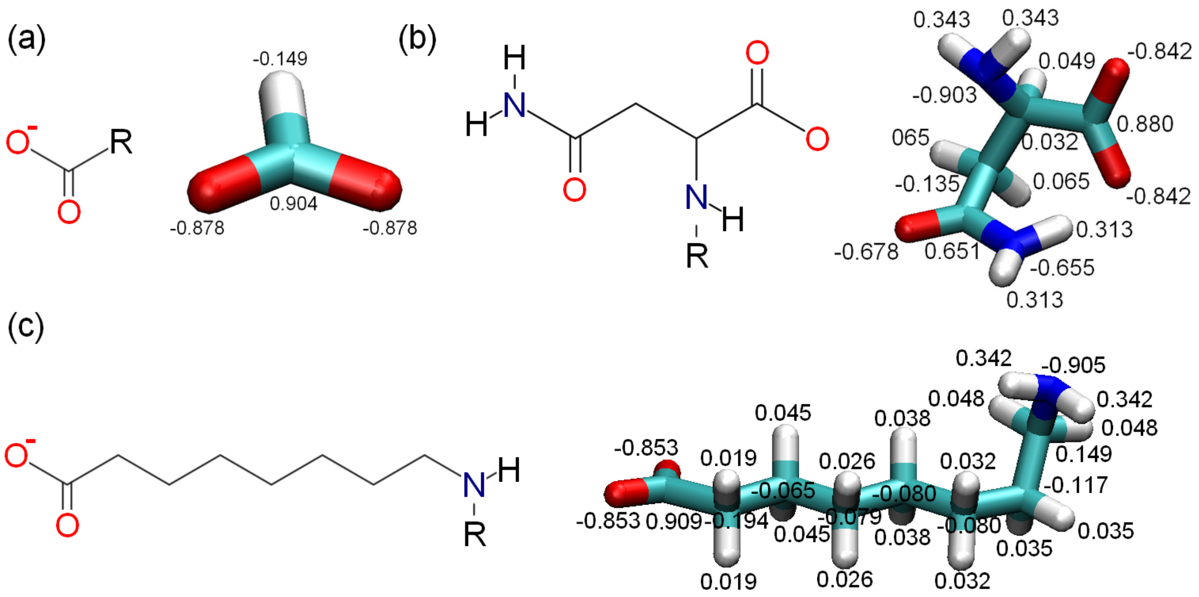

Figure S1. Chemical structure and charge distribution of functional groups **(a)** carboxyl (COO), **(b)** asparagine (Asn), and **(c)** 8-aminocaprylic acid (ACA). Hydrogen of functional group is deleted to bind carbon atom where functionalization takes place (denoted as R). (C: cyan, O: red, N: blue, H: white)

S2. Details of trajectory analysis

Hydrogen bonding: Electronegative atoms (O or N) of water and functional molecules are assumed to be involved in hydrogen bonding interactions with other water molecules. O and N atoms which are covalently bound to H are referred to as both donor and acceptor while O atoms which are not bound to H are acceptors. Accordingly, hydrogen bonding interactions between water-water and water-functional molecules are examined based on the geometric criteria (donor-acceptor distance ≤ 3.5 Å and donor-hydrogen-acceptor angle ≤ 30°).[1]

Interaction energy: Interaction energy between two molecules is calculated as the sum of intermolecular pairwise energy between all pairs of atoms. Interaction energy is contributed by Lennard-Jones 12-6 potential and Coulomb potential described below:

$$E = 4\varepsilon\left[\left(\frac{\sigma}{r}\right)^{12} - \left(\frac{\sigma}{r}\right)^{6}\right] \quad r<r_c \tag{S1}$$

ε is the van der Waals well depth, σ is the distance where the potential is zero, r is the distance between atoms, and r_c is the cutoff.

$$E = \frac{Cq_iq_j}{\epsilon r} \quad r<r_c \tag{S2}$$

C is the conversion factor, q_i, q_j are partial charges on the atoms, ϵ is the dielectric constant, r is the distance between atoms, and r_c is the cutoff.

Radial distribution function (RDF): The RDF**,** denoted as g(r), gives the probability of finding an atom at a distance r from the reference atom. The RDF analyses are carried out for different reference atoms such as terminal O or N atoms of functional groups as well as ions. Distance of water oxygens with respect to these atoms is calculated for every frame of the trajectory to estimate g(r). This analysis is performed via "measure gofr" algorithm integrated in VMD.

Potential of mean force (PMF): PMF for water molecules is calculated based on the density profile as follows:[2]

$$\mathrm{PMF}(z) = -kT\ln\left(\frac{\rho(z)}{\rho_{avg}}\right) \tag{S3}$$

where k is the Boltzmann constant, T is the temperature and ρ is the atom density.

S3. Analysis of water molecules confined in CNTs

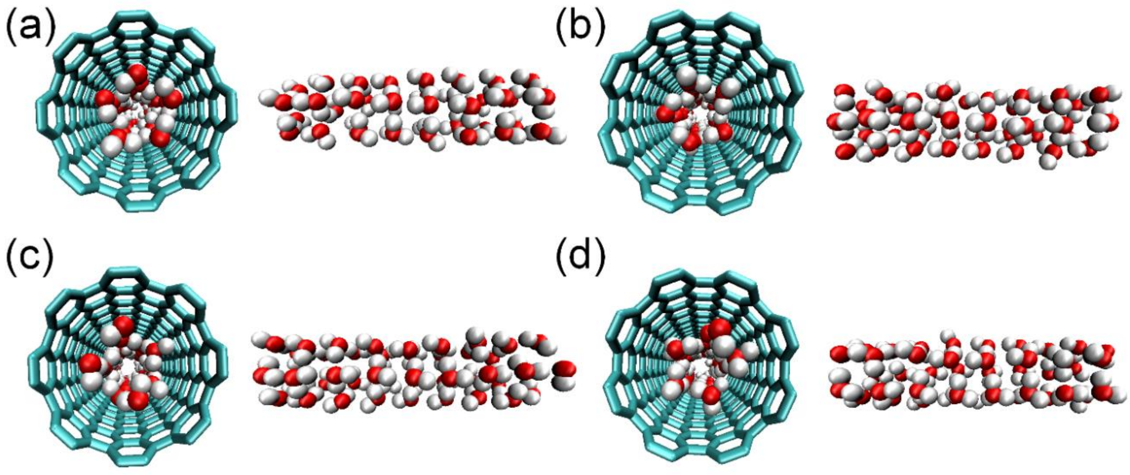

Figure S2. Five-molecule chain geometry of waters confined within the CNTs, **(a)** PRT, **(b)** COO, **(c)** ASN, and **(d)** ACA.

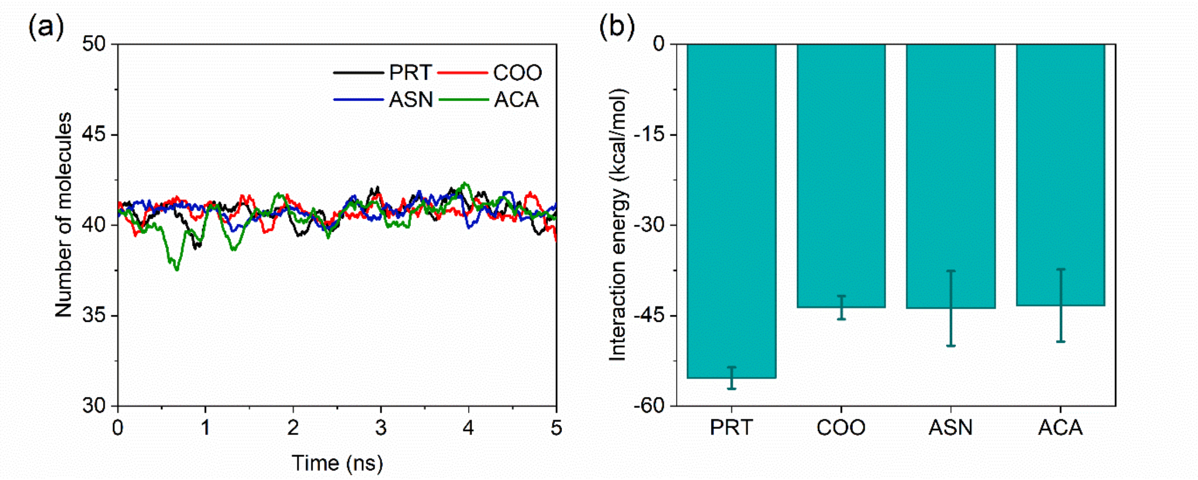

Figure S3. Water molecules confined within the CNTs, **(a)** number of molecules, and **(b)** time-averaged interaction energy per water molecule.

S4. Radial distribution functions

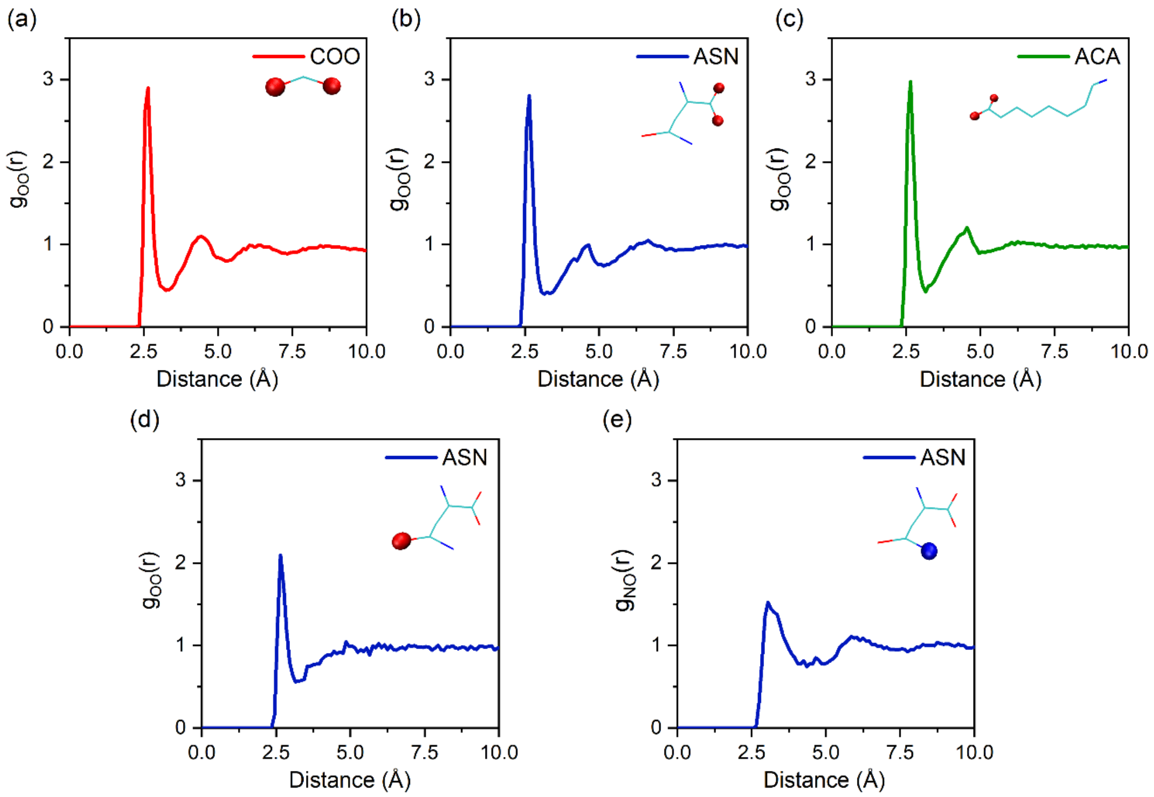

Figure S4. Radial distribution functions between water oxygens and **(a)** carboxylate oxygen of COO^-, **(b)** carboxylate oxygen of Asn backbone, **(c)** carboxylate oxygen of ACA, **(d)** amide oxygen of Asn side chain, and **(e)** amide nitrogen of Asn side chain.

S5. 2D water density maps

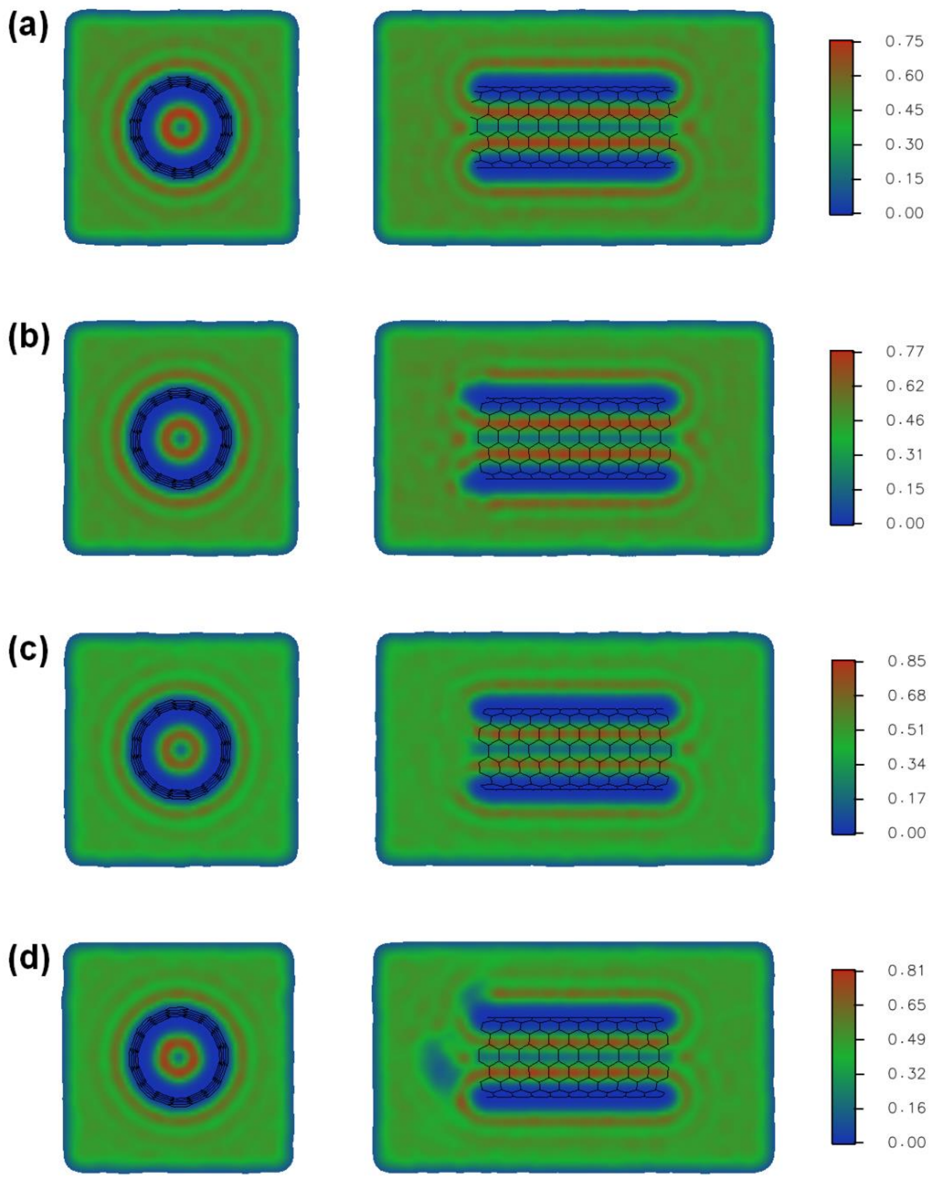

Figure S5. 2D-water density maps of **(a)** PRT, **(b)** COO, **(c)** ASN, and **(d)** ACA systems. XY plane croos-section (left) and YZ plane cross-section (right) are shown. Scale indicates atomic number density.

S6. Ion-water and ion-CNT interactions

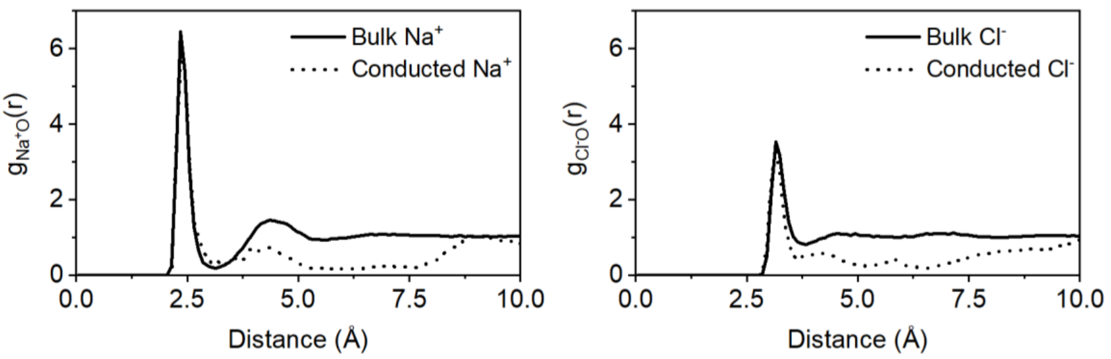

Figure S6. The RDFs between ions and water oxygens in PRT.

Table S1. Lennard-Jones 12-6 parameters of CNT carbons and ions taken from AMBER ff94 and ff99 force fields [3,4].

Atom	ε^a kcal·mol^{-1}	$r_0{}^b$ Å	σ^c Å
C	0.08600	1.9080	3.3997
Na	0.00277	1.8680	3.3284
Cl	0.10000	2.4700	4.4010

[a] van der Waals well depth, [b] van der Waals radius, [c] distance where the potential is zero with $\sigma=\frac{2r_0}{\sqrt[6]{2}}$

S7. Ion and water conductivities estimated from NEMD simulations

Table S2. Number of conducted molecules for each system. Three independent 40-ns-long NEMD simulations are carried out for each system. Average conductivity value for each system is shown in bold along with ± standard deviation. Force constant of 0.05 kcal·mol^{-1}·Å^{-1} is applied to ~360 molecules corresponding to ~140 MPa.

Membrane code	Functional group	Na^+ conductivity	Cl^- conductivity	H_2O conductivity
PRT_1	–	1	2	2632
PRT_2	–	0	3	2693
PRT_3	–	0	1	2689
		**0.33** ± 0.577	**2.00** ± 1.000	**2671** ± 34.1
COO_1	Carboxyl	3	0	2300
COO_2	Carboxyl	2	2	2244
COO_3	Carboxyl	2	0	2285
		**2.33** ± 0.577	**0.67** ± 1.155	**2276** ± 29.0
ASN_1	Asparagine	0	0	2189
ASN_2	Asparagine	2	1	2209
ASN_3	Asparagine	0	2	2052
		**0.67** ± 1.155	**1.00** ± 1.000	**2150** ± 85.5
ACA_1	8-aminocaprylic acid	0	0	1460
ACA_2	8-aminocaprylic acid	1	0	1375
ACA_3	8-aminocaprylic acid	2	0	1414
		**1.00** ± 1.000	**0.00** ± 0.000	**1416** ± 42.5

S8. Relation between water flux and hydrostatic pressure difference in PRT

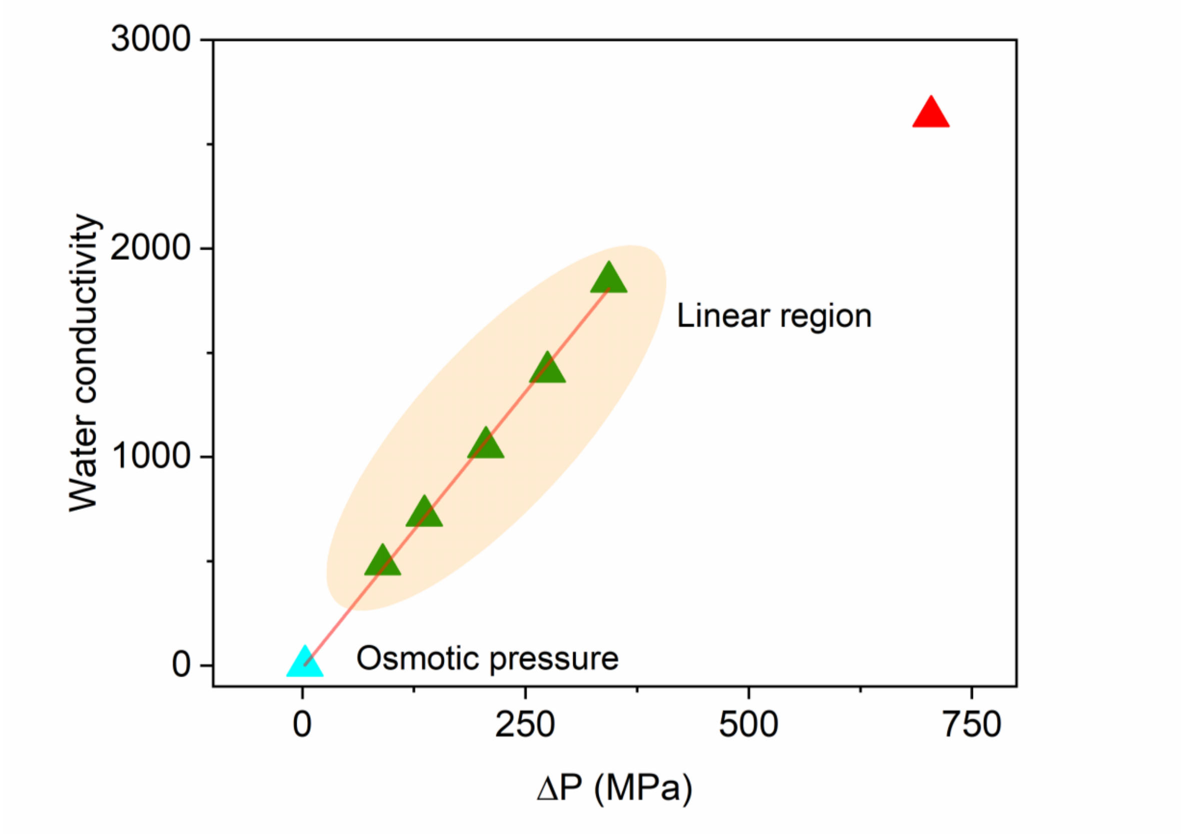

Figure S7. Number of conducted water molecules under different hydrostatic pressures from 10-ns-long simulations performed for PRT system. A linear relationship is found from ~90 to 340 MPa and gives R^2 value of 0.998 when intercepted with osmotic pressure (3.03 MPa). Note that ~700 MPa do not fit the linearity.

S9. Comparison of single channel water permeabilities

Based on the number of conducted water molecules, osmotic permeability coefficient, p_f ($cm^3 \cdot s^{-1}$) of CNTs is calculated as follows:[5]

$$p_f = \frac{j}{\Delta P} k_B T \tag{S4}$$

where j is the net water flux through a single channel ($molecules \cdot s^{-1}$), ΔP is the pressure difference, k_B is Boltzmann constant and T is the temperature. If available, single channel osmotic permeability coefficient reported in the study is given in **Table S3**. If not available, it is calculated using Equation S4.

Table S3. Single channel permeabilities of several water channels reported in the literature.

Ref.	Channel	Method[a]	Inner pore diameter[b] (nm)	Water conductivity ($molecules \cdot ns^{-1}$)	Pressure difference[c] (MPa)	Osmotic permeability coefficient ($cm^3 \cdot s^{-1}$)
[6]	wCNTP	E	1.35		2.97	5.90E-14
[6]	nCNTP	E	0.68		2.97	6.80E-13
[7]	AQP1	E	0.30		8.71	3.20E-13
[7]	AQPZ	E	0.28		8.71	1.80E-13
[8]	PAP[5]	E	0.50		11.6	1.00E-14
[9]	PAH[4]	E	0.30		5.79	1.10E-13
[10]	AQP1	S	0.30	3.36	195	7.10E-14
[11]	(8,8) SWCNT +8COO^-	S	1.09	14.0	246	2.34E-13
[11]	(8,8) SWCNT +4NH_3^+	S	1.09	22.1	246	3.70E-13
[12]	(20,0) SWCNT +2Zw	S	1.56	100	208	1.98E-12
[13]	(10,10) SWCNT +4-m-$CONH_2$[d]	S	1.35	124.7	200	2.57E-12
This work	(8,8) SWCNT +Asn	S	1.09	53.8	142	1.56E-12

SWCNT: Single-walled carbon nanotube, wCNTP: wide carbon nanotube porin, nCNTP: narrow carbon nanotube porin, AQP1: human aquaporin-1, AQPZ: *Escherichia coli* aquaporin, PAP[5]: peptide-appended pillar[5]arene, PAH[4]: peptide-appended hybrid[4]arene.

[a] The abbreviations E and D stand for experimental and simulation, respectively.

[b] For functionalized CNTs, diameter of its pristine form is given.

[c] For experimental studies, pressure difference is calculated as the osmotic pressure of test solution.

[d] m means functional molecule is added to interior surface of CNT.

S10. Characterization of f-CNTs via XPS

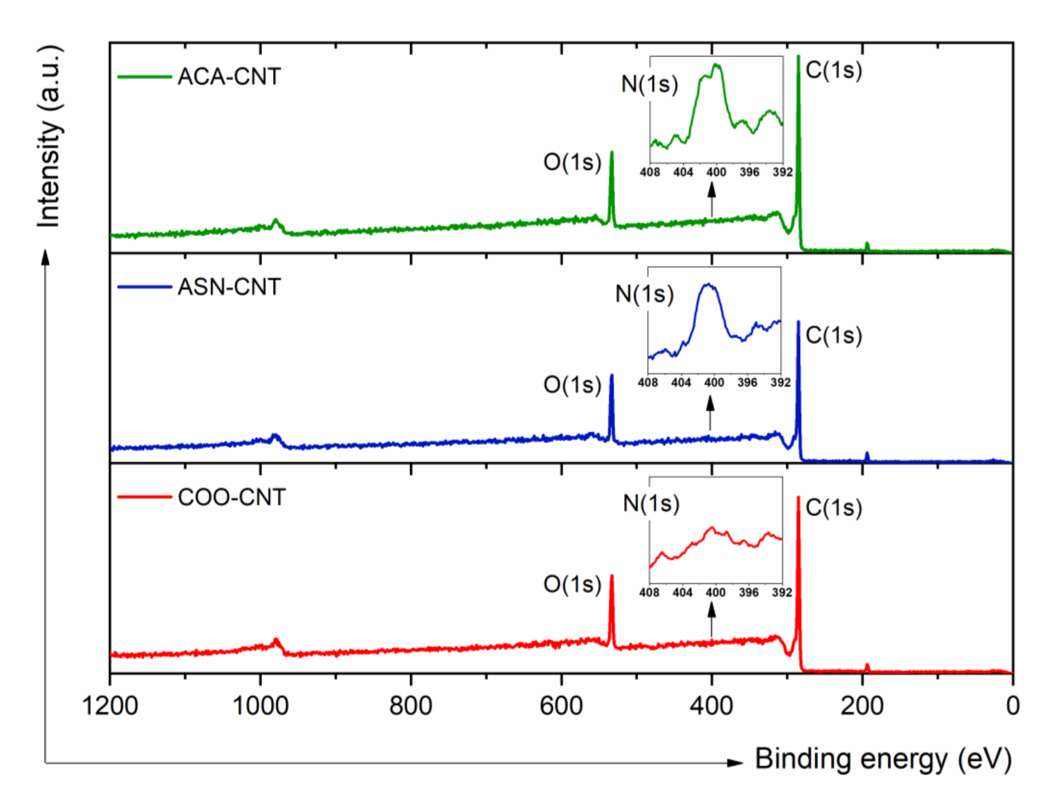

Figure S8. XPS survey spectra of f-CNTs (insets focus on deconvoluted high resolution N(1s) spectra).

Table S4. Atomic composition of functionalized CNTs obtained by XPS analysis.

CNT	C (1s) %	N (1s) %	O (1s) %
COO-CNT	89.10	-	10.90
ASN-CNT	86.37	0.79	12.84
ACA-CNT	89.60	0.54	9.86

S11. Characterization of TFC and TFN membranes

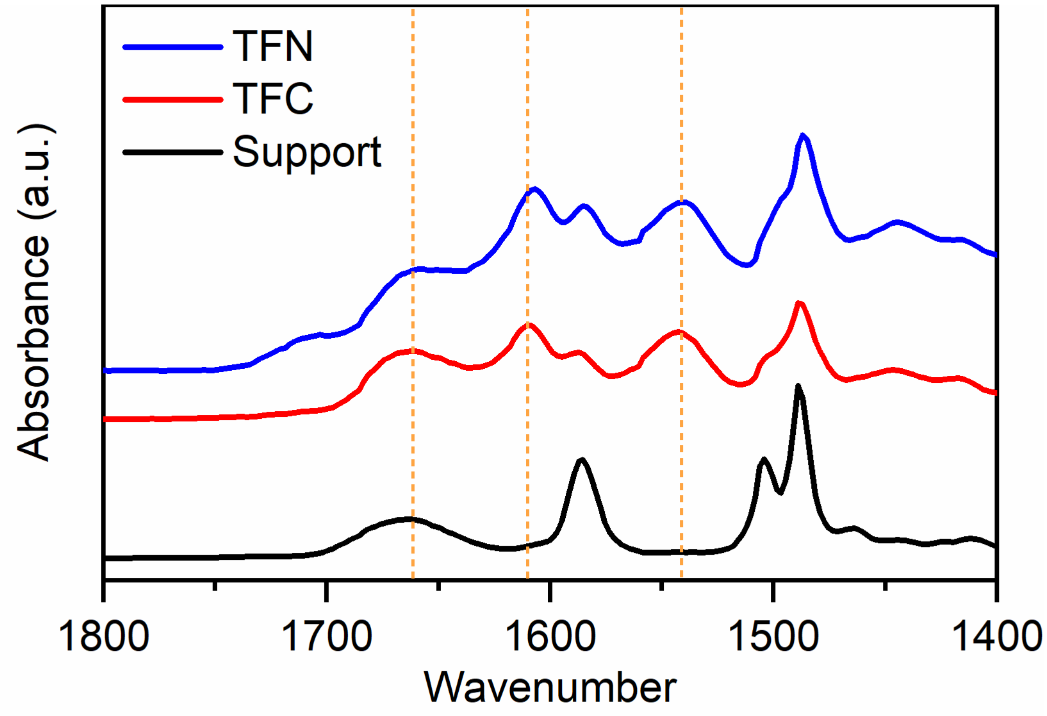

Figure S9. FTIR spectra of polysulfone support, TFC and COO-TFN which is very similar to ASN-TFN and ACA-TFN. PA characteristic absorption bands, 1660 cm^{-1} (C=O stretching in PA), 1542 cm^{-1} (C-N stretching in PA), and 1610 cm^{-1} (aromatic ring breathing in PA) appear in TFC and TFN membranes.

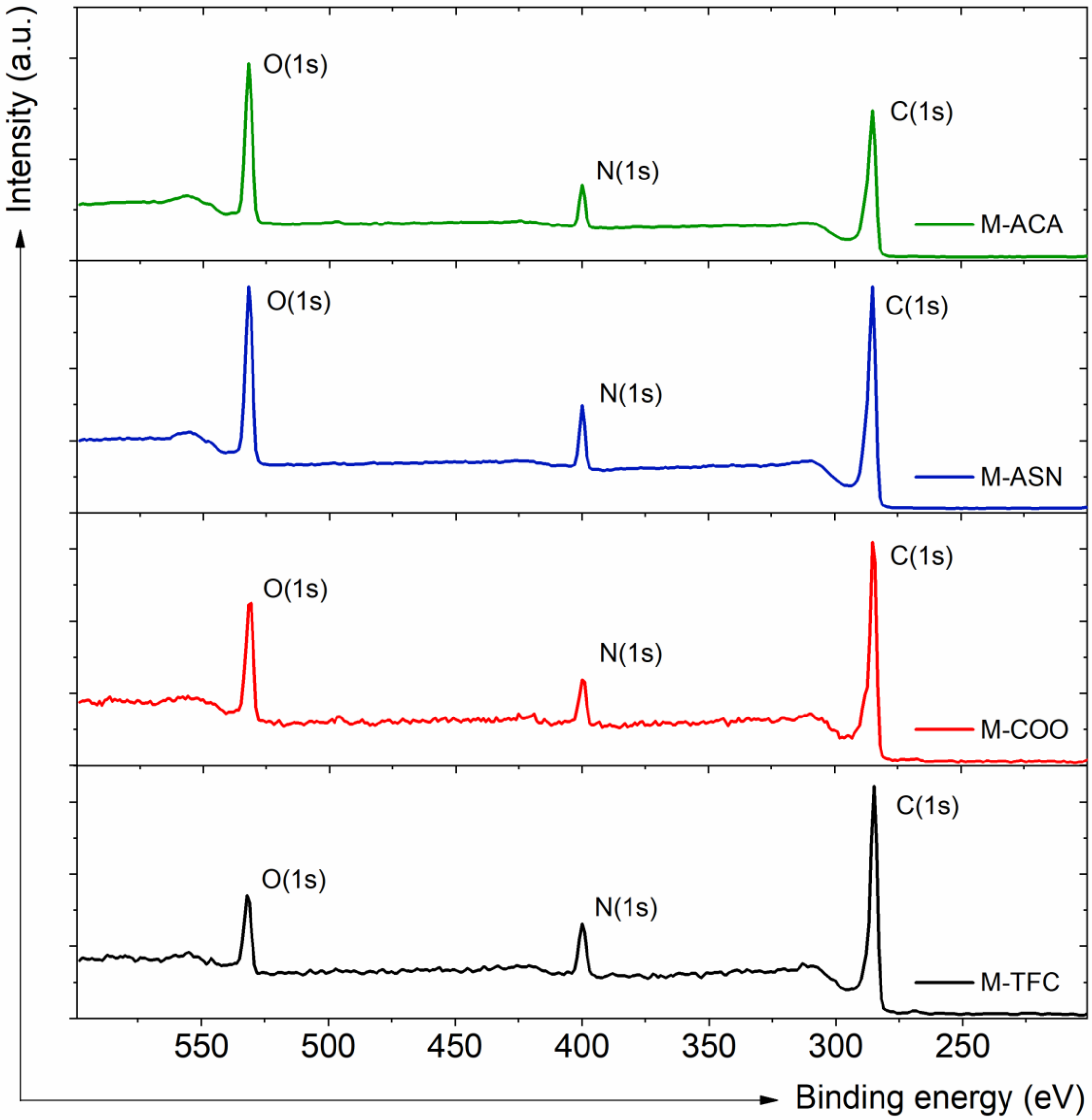

Figure S10. XPS survey spectra of TFC and TFN membranes embedding f-CNTs. (M-TFC and M-COO are taken from our previous work.[14])

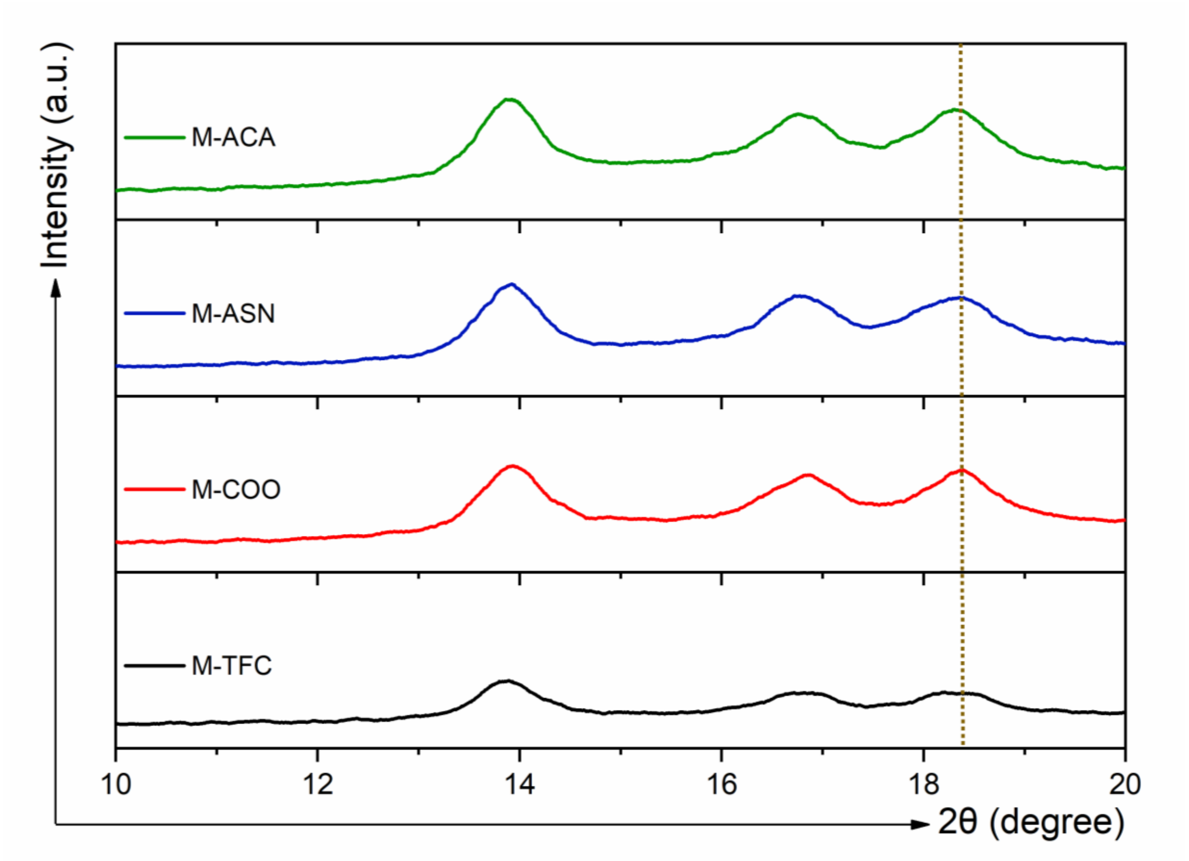

Figure S11. XRD measurements of TFC and TFN membranes embedding f-CNTs.

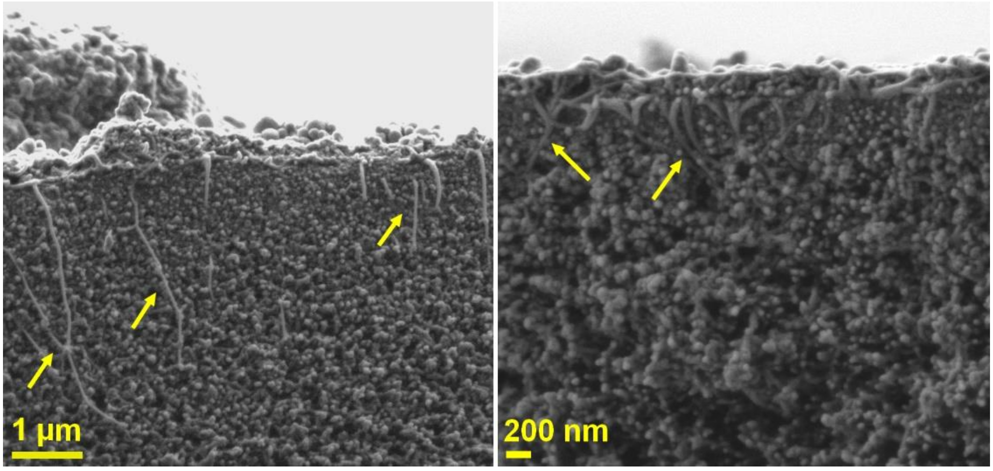

Figure S12. Detailed SEM images of M-ASN cross-section. Yellow arrows point out CNTs.

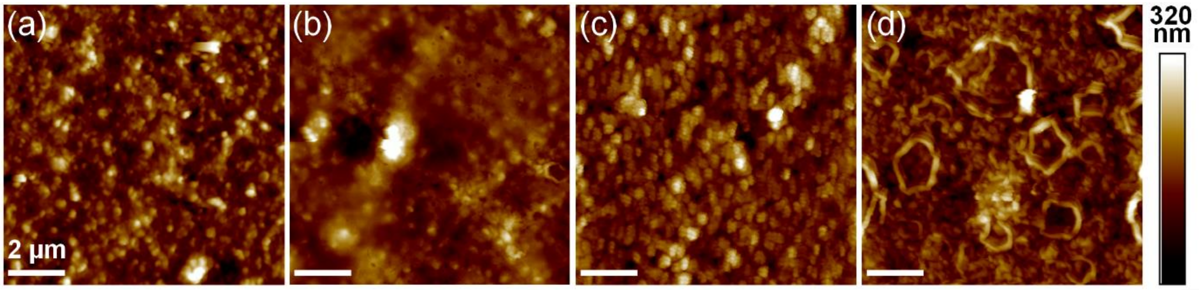

Figure S13. AFM surface images of **(a)** M-TFC, **(b)** M-COO, **(c)** M-ASN, and **(d)** M-ACA. Scale bars show 2 µm.

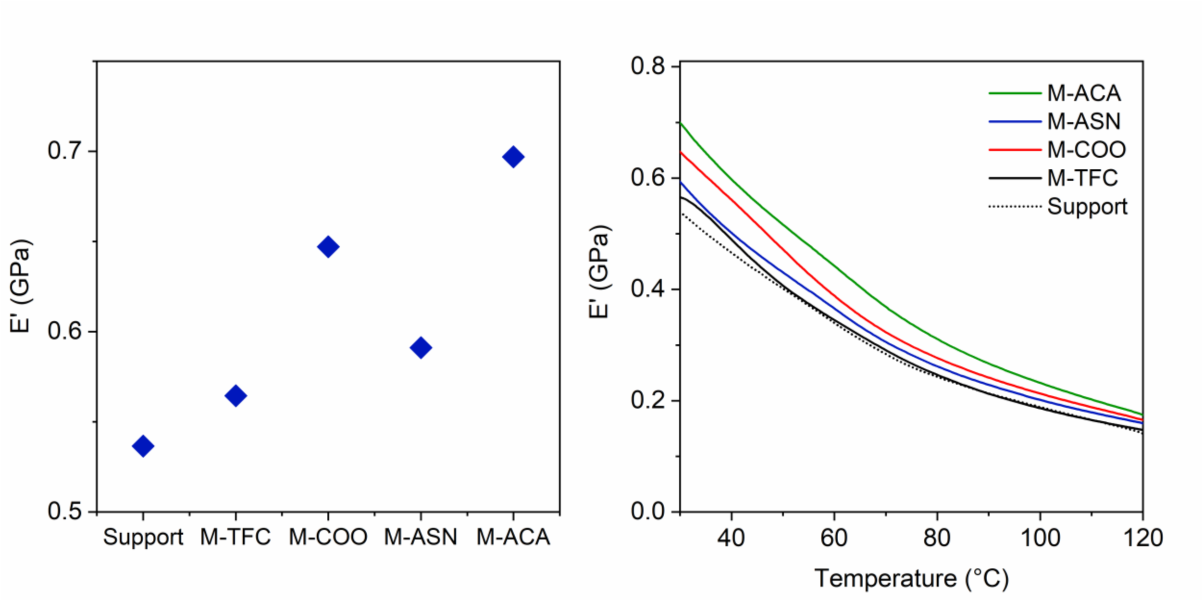

Figure S14. DMA analysis of TFC and TFN membranes **(a)** E' measured at 30°C and **(b)** change in storage moduli (E') as a function of temperature.

S12. Separation performance of TFC and TFN membranes

Table S5. Separation performance of our TFC and TFN membranes. Data for three independent membrane replicas prepared under identical conditions is reported. Average NaCl rejection and pure water flux for each membrane type is shown in bold along with ± standard deviation.

Membrane code	Functional group	NaCl rejection, %	Pure water flux, LMH
M-TFC-1	–	98.3	28.5
M-TFC-2	–	97.8	15.1
M-TFC-3	–	97.3	19.8
		**97.8** ± 0.50	**21.1** ± 6.80
M-COO-1	Carboxyl	98.2	33.3
M-COO-2	Carboxyl	98.4	17.6
M-COO-3	Carboxyl	98.4	21.2
		**98.3** ± 0.12	**24.0** ± 8.22
M-ASN-1	Asparagine	98.0	32.9
M-ASN-2	Asparagine	98.3	21.2
M-ASN-3	Asparagine	98.5	25.4
		**98.3** ± 0.25	**26.5** ± 5.93
M-ACA-1	8-aminocaprylic acid	98.0	20.1
M-ACA-2	8-aminocaprylic acid	95.9	23.5
M-ACA-3	8-aminocaprylic acid	97.6	21.3
		**97.2** ± 1.12	**21.6** ± 1.72

S13. Equations for the calculation of water and salt permeability coefficients

Water permeability coefficient, A, is calculated from the equation,[15]

$$A = \frac{J_w}{\left(\Delta P - \Delta \pi_b\right)} \tag{S5}$$

where J_w is the water flux, while ΔP and $\Delta \pi_b$ are hydraulic pressure difference and bulk osmotic pressure difference across the membrane, respectively. If the pure water flux is reported in the study, equation becomes,

$$A = \frac{J_w}{\Delta P} \tag{S6}$$

Salt permeability coefficient, B, is calculated as,

$$B = J_w \left(\frac{1}{R} - 1 \right) \tag{S7}$$

where R is the apparent salt rejection of the membrane.

Permselectivity is defined as,

$$permselectivity = A/B \tag{S8}$$

whereas improvement in permselectivity is calculated as,

$$improvement = \frac{\left(permselectivity\right)_{TFN} - \left(permselectivity\right)_{TFC}}{\left(permselectivity\right)_{TFC}} \times 100 \tag{S9}$$

S14. Comparison of experimental performance with the literature

Table S6. Details of AQP/PA and CNT/PA studies presented in Table 2.

Ref.[a]	Filler	Testing conditions[b]	Water flux[c], LMH	Salt rejection, %	Water perm. coefficient (A), $LMH \cdot bar^{-1}$	Salt perm. coefficient (B), $LMH \cdot bar^{-1}$	Perm-selectivity (A/B)
This work	TFC	15.5 bar, 2000 ppm	21.1	97.8	1.36	0.47	2.87
	Asn func. SWCNTs, D: 1 nm		26.5	98.3	1.71	0.46	3.73
[38]	TFC	36.5 bar, 2500 ppm	11.7	97.6	0.34	0.29	1.18
	Zwitterion func. SWCNTs, D: 1.5 nm		48.5	98.6	1.41	0.69	2.05
[41]	TFC	15 bar, 2000 ppm	42.0	95	2.80	2.21	1.27
	Amine func. MWCNTs, D: 5-20 nm		56.0	97.3	3.73	1.55	2.40
[42]	TFC	15.5 bar, 2000 ppm	36.0	97.9	2.60	0.77	3.37
	Polyacrylamide func. MWCNTs, D: 20-30 nm		48.4	98.9	3.50	0.54	6.50
[64]	TFC	5 bar, 584 ppm	16.0	96.3	3.54	0.61	5.75
	AQPZ containing proteoliposomes		18.1	96.9	4.00	0.58	6.92
[65]	TFC	10 bar, 584 ppm	26.8	96.5	2.81	0.97	2.90
	AQP containing proteoliposomes		39.2	97.1	4.12	1.17	3.52
[66]	TFC	5 bar, 500 ppm	21.0	92.7	4.56	1.65	2.76
	AqpZ-containing polymersomes		29.2	93.5	6.35	2.03	3.12
[67]	TFC	55 bar, 32000 ppm	11.2	99.0	0.40	0.11	3.52
	AQP-containing DOPC proteoliposomes		21.0	99.0	0.75	0.21	3.52

a References in manuscript is given.
b Hydraulic pressure difference and NaCl concentration of the feed solution.
c Pure or salt water flux is reported. Note that this is considered in the calculation of permeability coefficients.
The abbreviation D stands for diameter.

S15. Description of the animated video

A part of the 40-ns-long NEMD trajectory of ASN system is shown in the animation. Asn-functionalized CNT and surrounding graphene walls are depicted in stick representation while water molecules (cyan), Na^{+} (green), and Cl^{-} (yellow) are depicted as spheres.

References

(1) Hummer, G.; Rasaiah, J. C.; Noworyta, J. P. Water Conduction through the Hydrophobic Channel of a Carbon Nanotube. *Nature* **2001**, *414* (6860), 188–190.

(2) Corry, B. Designing Carbon Nanotube Membranes for Efficient Water Desalination. *J. Phys. Chem. B* **2008**, *112* (5), 1427–1434.

(3) Bayly, C. I.; Merz, K. M.; Ferguson, D. M.; Cornell, W. D.; Fox, T.; Caldwell, J. W.; Kollman, P. A.; Cieplak, P.; Gould, I. R.; Spellmeyer, D. C. A Second Generation Force Field for the Simulation of Proteins, Nucleic Acids, and Organic Molecules. *J. Am. Chem. Soc.* **1995**, *117* (19), 5179–5197.

(4) Wang, J.; Cieplak, P.; Kollman, P. A. How Well Does a Restrained Electrostatic Potential (RESP) Model Perform in Calculating Conformational Energies of Organic and Biological Molecules? *J. Comput. Chem.* **2000**, *21* (12), 1049–1074.

(5) Velioğlu, S.; Karahan, H. E.; Goh, K.; Bae, T.-H. H. T.; Chen, Y.; Chew, J. W. Metallicity-Dependent Ultrafast Water Transport in Carbon Nanotubes. *Small* **2020**, *16* (25), 1907575.

(6) Tunuguntla, R. H.; Henley, R. Y.; Yao, Y.-C.; Pham, T. A.; Wanunu, M.; Noy, A. Enhanced Water Permeability and Tunable Ion Selectivity in Subnanometer Carbon Nanotube Porins. *Science* **2017**, *357* (6353), 792–796.

(7) Horner, A.; Zocher, F.; Preiner, J.; Ollinger, N.; Siligan, C.; Akimov, S. A.; Pohl, P. The Mobility of Single-File Water Molecules Is Governed by the Number of H-Bonds They May Form with Channel-Lining Residues. *Sci. Adv.* **2015**, *1* (2), e1400083.

(8) Shen, Y.; Si, W.; Erbakan, M.; Decker, K.; De Zorzi, R.; Saboe, P. O.; Kang, Y. J.; Majd, S.; Butler, P. J.; Walz, T.; et al. Highly Permeable Artificial Water Channels That Can Self-Assemble into Two-Dimensional Arrays. *Proc. Natl. Acad. Sci.* **2015**, *112* (32), 9810–9815.

(9) Song, W.; Joshi, H.; Chowdhury, R.; Najem, J. S.; Shen, Y. xiao; Lang, C.; Henderson, C. B.; Tu, Y.-M. M.; Farell, M.; Pitz, M. E.; et al. Artificial Water Channels Enable Fast and Selective Water Permeation through Water-Wire Networks. *Nat. Nanotechnol.* **2020**, *15* (1), 73–79.

(10) Zhu, F.; Tajkhorshid, E.; Schulten, K. Theory and Simulation of Water Permeation in Aquaporin-1. *Biophys. J.* **2004**, *86* (1), 50–57.

(11) Corry, B. Water and Ion Transport through Functionalised Carbon Nanotubes: Implications for Desalination Technology. *Energy Environ. Sci.* **2011**, *4* (3), 751–759.

(12) Chan, W. F.; Chen, H. Y.; Surapathi, A.; Taylor, M. G.; Shao, X.; Marand, E.; Johnson, J. K. Zwitterion Functionalized Carbon Nanotube/Polyamide Nanocomposite Membranes for Water Desalination. *ACS Nano* **2013**, *7* (6), 5308–5319.

(13) Li, Q.; Yang, D.; Shi, J.; Xu, X.; Yan, S.; Liu, Q. Biomimetic Modification of Large Diameter Carbon Nanotubes and the Desalination Behavior of Its Reverse Osmosis Membrane. *Desalination* **2016**, *379*, 164–171.

(14) Güvensoy-Morkoyun, A.; Kürklü-Kocaoğlu, S.; Yıldırım, C.; Velioğlu, S.; Karahan, H. E.; Bae, T.-H.; Tantekin-Ersolmaz, Ş. B. Carbon Nanotubes Integrated into Polyamide

Membranes by Support Pre-Infiltration Improve the Desalination Performance. *Carbon* **2021**, *185*, 546–557.

(15) Yang, Z.; Guo, H.; Tang, C. Y. The Upper Bound of Thin-Film Composite (TFC) Polyamide Membranes for Desalination. *J. Memb. Sci.* **2019**, *590*, 117297.
